# PINK1-mediated Drp1^S616^ phosphorylation modulates synaptic development and plasticity via promoting mitochondrial fission

**DOI:** 10.1038/s41392-022-00933-z

**Published:** 2022-04-15

**Authors:** Qingtao Gao, Runyi Tian, Hailong Han, Jesse Slone, Caifang Wang, Xiao Ke, Tongmei Zhang, Xiangyu Li, Yuhong He, Panlin Liao, Fang Wang, Ye Chen, Shiqing Fu, Kexuan Zhang, Fangfang Zeng, Yingxuan Yang, Zhuo Li, Jieqiong Tan, Jiada Li, Youming Lu, Taosheng Huang, Zhonghua Hu, Zhuohua Zhang

**Affiliations:** 1 Institute of Molecular Precision Medicine, Xiangya Hospital, Key Laboratory of Molecular Precision Medicine of Hunan Province and Center for Medical Genetics, Hunan Key Laboratory of Medical Genetics, Central South University, Changsha, Hunan 410008 China; 2 Department of Pediatrics, Jacobs School of Medicine and Biomedical Sciences, University at Buffalo, Buffalo, New York 14203 USA; 3 Department of Physiology, School of Basic Medicine and Tongji Medical College, Huazhong University of Science and Technology, Wuhan, Hubei 430030 China; 4 The Institute for Brain Research, Collaborative Innovation Center for Brain Science, Huazhong University of Science and Technology, Wuhan, Hubei 430030 China; 5 Hunan Key Laboratory of Animal Models for Human Diseases, School of Life Sciences, Central South University, Changsha, Hunan 410008 China; 6 Department of Neurosciences, University of South China Medical School, Hengyang, Hunan 421001 China; 7 National Clinical Research Center for Geriatric Disorders, Xiangya Hospital, Central South University, Changsha, Hunan 410008 China; 8 Department of Critical Care Medicine, Hunan Provincial Clinical Research Center for Critical Care Medicine, Xiangya Hospital, Central South University, Changsha, Hunan 410008 China

**Keywords:** Cellular neuroscience, Cell biology

## Abstract

Dynamic change of mitochondrial morphology and distribution along neuronal branches are essential for neural circuitry formation and synaptic efficacy. However, the underlying mechanism remains elusive. We show here that *Pink1* knockout (KO) mice display defective dendritic spine maturation, reduced axonal synaptic vesicles, abnormal synaptic connection, and attenuated long-term synaptic potentiation (LTP). Drp1 activation via S616 phosphorylation rescues deficits of spine maturation in *Pink1* KO neurons. Notably, mice harboring a knockin (KI) phosphor-null *Drp1*^*S616A*^ recapitulate spine immaturity and synaptic abnormality identified in *Pink1* KO mice. Chemical LTP (cLTP) induces Drp1^S616^ phosphorylation in a PINK1-dependent manner. Moreover, phosphor-mimetic Drp1^S616D^ restores reduced dendritic spine localization of mitochondria in *Pink1* KO neurons. Together, this study provides the first in vivo evidence of functional regulation of Drp1 by phosphorylation and suggests that PINK1-Drp1^S616^ phosphorylation coupling is essential for convergence between mitochondrial dynamics and neural circuitry formation and refinement.

## Introduction

Neurons are highly polarized cells that consist of numerous branches including dendrites and axons. The distribution and transportation of organelles along neural branches are of great importance for neuronal development and function. Mitochondria are vital organelles asymmetrically distributed in neuronal compartments.^1^ They are under dynamic morphological change and active trafficking during neuron development or in response to neuron activity.^2^ Mitochondrial dynamics, including morphological change and distribution, have been recognized as crucial mechanisms to support their functions in regulating energy supply, Ca^2+^ homeostasis, neurotransmitter synthesis, ROS generation, apoptosis, metabolism, and other aspects of neuron physiology.^2^ These dynamic modifications also provide a capability for spatiotemporal regulation of local cellular events to adapt to the extreme complexity of neuronal compartments including axons, dendrites, and synapses.

Mitochondrial morphology and functions are regulated by constant fusion and fission dynamics and motility.^1,3^ Fusion is triggered by several GTPases, for example, Mfn1 and Mfn2 at the outer membrane, and Opa1 at the inner membrane. Another GTPase in the dynamin family, Drp1 (dynamin-related protein-1) is a key component to execute fission at the outer membrane. Drp1 is recruited to the mitochondria membrane by Fis1 and MFF to facilitate fission. Notably, posttranslational modifications to Drp1 through phosphorylation, SUMOylation, or S-nitrosylation are important for its fission activity in vitro.^3^ Meanwhile, trafficking and anchoring of mitochondria are regulated by microtubule or actin-based proteins.^4^ Together, these molecular machineries coordinately determine the size, content, shape, and localization of mitochondria, therefore are crucial for proper mitochondrial function in neurons.

Synapse is the key contact site between neurons. Numerous molecular and cellular processes consuming energy occur locally at or close to synapses. Mitochondrial dynamics is critical at these sites to support the wiring of synaptic circuitry and synaptic plasticity.^5–7^ Accumulating evidence suggests that alteration of mitochondrial size or disruption of key fission molecule Drp1 result in abnormalities of synaptic development, plasticity, and function. For instance, deletion of *Drp1* in mice leads to aggerated mitochondria and reduced expression of synaptophysin.^8^ *Drp1* knockout in forebrain excitatory neuron results in enlargement of mitochondria and decreased mitochondrial-derived ATP in the axon, impaired synaptic transmission, and hippocampus-dependent memory.^9^ *Drp1* knockdown in D1-Medium spiny neuron in nucleus accumbens increases the number of large mitochondria and blocks cocaine-seeking after drug exposure.^10^ Moreover, expression of Drp1-K38A, a dominant-negative form of Drp1, in cultured hippocampal neurons decreases mitochondrial content and dendritic spine numbers and blocks neuron activity-induced increase of postsynaptic puncta.^5^ In hippocampal neurons, activity-dependent enhancement of mitochondrial motility and entering into dendritic protrusions are promoted by Drp1 overexpression while inhibited by Drp1-K38A, suggesting profound synaptic influences of Drp1-mediated mitochondrial dynamics.^5^

The fission activity of Drp1 is regulated by different posttranslational modifications.^11^ Drp1 phosphorylation at different serine sites, such as 616, 637, 656, plays important role in mitochondria dynamics.^12–15^ Despite several in vitro studies implicating important roles of Drp1 phosphorylation in synapse development and plasticity,^6,14^ they are yet to be verified in vivo.

PTEN-induced kinase 1 (PINK1) encodes a serine/threonine kinase targeted to mitochondria.^16^ PINK1 promotes mitochondrial fission in flies.^17–20^ In mammalian cells, PINK1 has variable effects on fission in a different cell context.^21,22^ We recently demonstrated that PINK1 directly phosphorylates Drp1^S616^ to regulate mitochondrial fission.^23^

In this study, we aimed to investigate the roles of PINK1-mediated Drp1^S616^ phosphorylation in the central nervous system. Our results demonstrate increased detection of elongated mitochondria in neurons in the hippocampus and cortex of *Pink1* KO mice. In these mice, mitochondria are less frequently found in dendritic spines and presynaptic boutons during excitatory synapse maturation. Localization of mitochondria in dendritic spines upon LTP induction is suppressed in *Pink1* KO neurons. *Drp1*^*S616A*^ KI mice, bearing phosphor-null mutation of Drp1, phenocopy *Pink1* KO mice in defects of synaptic development and function. Notably, *Drp1*^*S616A*^ KI mice exhibit inhibited hippocampal-dependent learning and memory. Together, our results suggest that PINK1-mediated Drp1^S616^ phosphorylation plays an essential role in regulating mitochondrial dynamics, therefore, contributing to synapse maturation, synaptic transmission and plasticity, and learning and memory. This study uncovered a novel kinase/substrate cassette that couples mitochondrial dynamics with the development and function of neural circuitry.

## Results

### PINK1 regulates dendritic spine morphogenesis and excitatory synapse formation

To investigate the physiological function of PINK1 on mitochondrial dynamics during synapse development, we labeled mitochondria and cultured neurons derived from *Pink1* KO and wild-type (WT) mice with MitoGFP and DsRed, respectively. Length and number of dendritic mitochondria at days in vitro (DIV) 18, a critical period of synapse maturation, were examined and quantified. The mean mitochondrial length was 3.783 ± 0.215 µm in WT hippocampal neurons, while it increased to 10.48 ± 0.647 µm in *Pink1* KO neurons. In contrast, the density of dendritic mitochondria was decreased in *Pink1* KO neurons (Supplementary Fig. S1a, b, 16.86 ± 0.744 mitochondria per 100 µm in WT neurons; 9.15 ± 0.452 mitochondria per 100 µm in *Pink1* KO neurons). Likewise, the length of axonal mitochondria was also longer in *Pink1* KO neurons than that in WT neurons (Supplementary Fig. S1a, b). Results indicate that mitochondria are longer in size but less in number in *Pink1* KO neurons than those in WT neurons. Next, neuronal mitochondria in the hippocampus and cortex were analyzed in vivo using transmission electron microscopy (TEM). Length of dendritic mitochondria increased ~1.5-fold in *Pink1* KO neurons at age of 8 weeks compared to that in WT neurons (Supplementary Fig. S1c, d, 0.87 ± 0.050 µm in WT hippocampal neurons; 1.28 ± 0.100 µm in *Pink1* KO hippocampal neurons. Supplementary Fig. S1e, f, 0.92 ± 0.071 µm in WT cortical neurons; 1.67 ± 0.144 µm in *Pink1* KO cortical neurons). Interestingly, mitochondria were visualized in ~16.75% presynaptic boutons in WT neurons, while only in ~11.75% in *Pink1* KO neurons (Supplementary Fig. S1g, h). 88.94% of WT boutons had mitochondria with a length less than 0.5 µm. In contrast, *Pink1* KO boutons showed an increased number of large mitochondria (0.5–1 µm in length) compared to WT boutons (Supplementary Fig. S1g, h, ~25.69% in *Pink1* KO neurons, ~11.06% in WT neurons). Together, dendritic and axonal mitochondrial fission are defective in *Pink1* KO neurons.

**Fig. 1:**
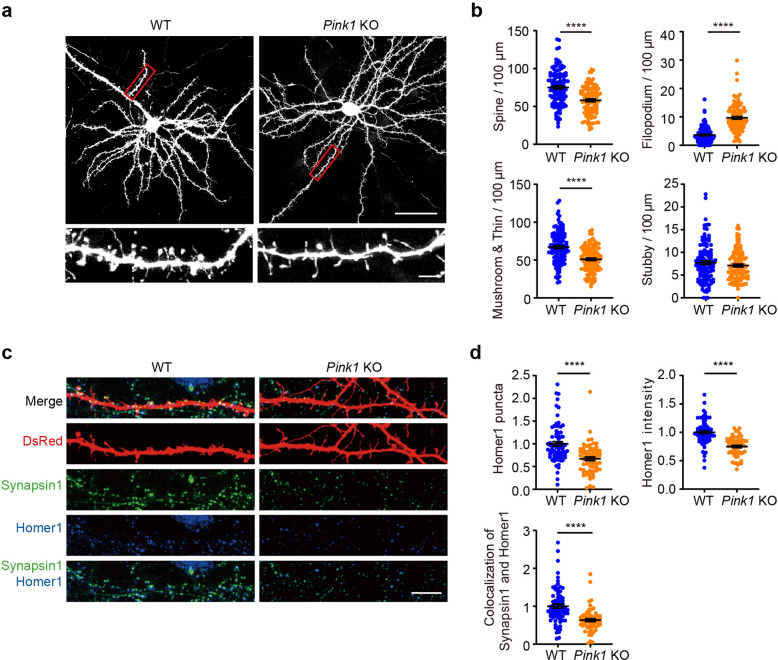
PINK1 regulates dendritic spine development and enrichment of synaptic proteins. **a** Cortical neurons (DIV 6–8) derived from WT or *Pink1* KO mice were transfected with plasmid encoding EGFP and imaged at DIV 18. Representative images of neurons with low magnification (top, Bar = 50 μm) and neurites with higher magnification of gated areas (bottom, Bar = 5 μm) were shown. **b** Quantification of the density of dendritic protrusions and proportion of different types of protrusions for experiments presented in **a**. *n* = 116 and 109 dendrites for WT and *Pink1* KO from three independent experiments, respectively. **c** Cortical neurons (DIV 6–8) derived from WT or *Pink1* KO mice were transfected with DsRed plasmid followed by staining at DIV 18. Representative images of neurons were stained with antibodies against Homer1 (blue) or Synapsin1 (green). Bar = 10 μm. **d** Quantification of normalized synaptic puncta density or fluorescence intensity of dendritic Homer1 and colocalization of Synapsin1 and Homer1 for experiments shown in **c**. *n* = 76 and 65 dendrites for WT and *Pink1* KO from three independent experiments, respectively. All data were normalized with the mean value of WT dendrites in each repeat. ****P < 0.0001. Student’s *t*-test

We next studied the roles of PINK1 in the development of dendritic spines. Cortical or hippocampal neuronal cultures derived from WT or *Pink1* KO mice were quantified for spine number of secondary dendrites at DIV 18. Dendritic spines are classified into three groups based on their morphology, including mushroom, thin, and stubby spines.^24^ Both mushroom and thin spines have enlarged heads and constricted necks that are difficult to distinguish, we, therefore, combine them into a mushroom/thin spine group in this study. Results revealed that the density of total spines was significantly lower in *Pink1* KO cortical neurons than that in WT control neurons at DIV 18 (Fig. 1a, b, 75.00 ± 2.145 spines/100 µm in WT neurons; 58.07 ± 1.772 spines/100 µm in *Pink1* KO neurons). Notably, a decrease in density of mushroom/thin spines (Fig. 1a, b, 67.27 ± 1.989 spines/100 µm in WT neurons; 50.96 ± 1.62 spines/100 µm in *Pink1* KO neurons), but not stubby spines (Fig. 1a, b, 7.73 ± 0.401 spines/100 µm in WT neurons; 7.11 ± 0.312 spines/100 µm in *Pink1* KO neurons) was observed in *Pink1* KO cortical neurons comparing to that of WT control neurons. In contrast, the density of filopodium was increased in *Pink1* KO neurons (Fig. 1a, b, 3.62 ± 0.253 protrusions/100 µm in WT neurons; 9.65 ± 0.451 protrusions/100 µm in *Pink1* KO neurons). Results indicate immaturity of spines of cortical neurons derived from *Pink1* KO mice.^25,26^ Consistently, immature spines were observed in cultured *Pink1* KO hippocampal neurons (Supplementary Fig. S2a, b). Quantification of excitatory synapses by examining colocalized Homer1, a protein enriched in the postsynaptic density of excitatory synapse, and presynaptic Synapsin1 revealed that synapse density in cultured *Pink1* KO cortical neurons was lower than that in WT control neurons at DIV 18 (Fig. 1c, d). The fluorescence intensity of postsynaptic protein Homer1 was reduced in *Pink1* KO dendrites compared to WT dendrites, suggesting a decreased level of Homer1 expression at synapses (Fig. 1d). However, dendritic length and number of intersections were not changed in *Pink1* KO neurons compared to WT neurons (Supplementary Fig. S3a, b). Thus, PINK1 selectively regulates dendritic spine morphogenesis and excitatory synapse formation.

### *Pink1* KO mice exhibit abnormal hippocampal and cortical circuits

**Fig. 2:**
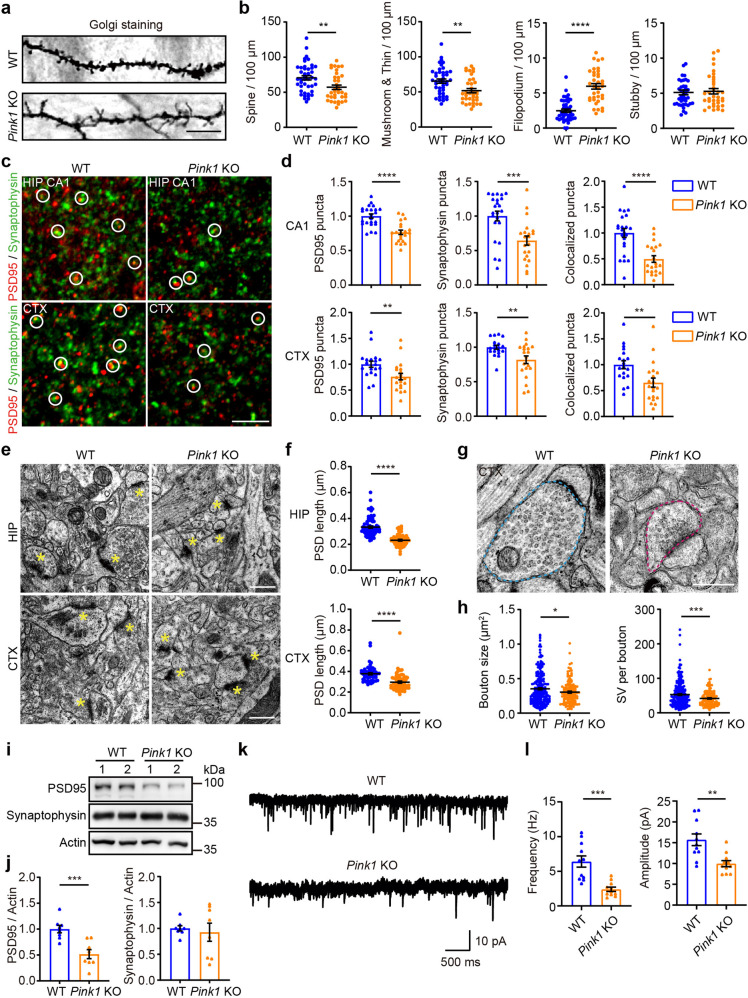
PINK1 regulates excitatory neuronal connections in the cortex and hippocampus. **a** Representative images of Golgi staining of somatosensory cortex layer 2/3 neurons from P14 WT and *Pink1* KO mice. Bar = 10 μm. **b** Quantitation of protrusion density at secondary apical dendrites of neurons for experiments presented in **a**. *n* = 43 and 36 dendrites from three WT and three *Pink1* KO mice, respectively. **c** Representative images of PSD95 and synaptophysin in 8-week-old WT or *Pink1* KO mice. Hippocampal CA1 stratum radiatum (HIP CA1) or somatosensory cortex layer 2/3 (CTX) were stained with PSD95 (red) and synaptophysin (green) and imaged. White circles indicate colocalized PSD95 and synaptophysin puncta. Bar = 5 μm. **d** Quantification of normalized synaptic puncta density for experiments presented in **c** using Imaris software. Puncta were reconstructed using Imaris “spot” function. Colocalized PSD95 and synaptophysin puncta were calculated. Four to five brain sections of each mouse from four WT and four *Pink1* KO mice were analyzed. **e** Representative TEM images of hippocampus CA1 (HIP CA1) and somatosensory cortex (CTX) of 8-week-old WT or *Pink1* KO mice. Synapses are marked by asterisks. Bar = 0.5 μm. **f** Quantitation of PSD length of experiments presented in **e**. HIP: *n* = 75 sections from three WT mice, *n* = 65 sections from three *Pink1* KO mice; CTX: *n* = 54 and 72 sections from three WT and three *Pink1* KO mice, respectively. **g** Representative TEM images of presynaptic boutons in the somatosensory cortex of 8-week-old WT and *Pink1* KO mice. Blue dash-line frame: presynaptic bouton with mitochondria. Magenta dash-line frame: presynaptic bouton without mitochondria. Bar = 0.5 μm. **h** Quantitation of bouton size and the number of synaptic vesicles per bouton of experiments presented in **g**. *n* = 238 and 143 synapses from three WT and two *Pink1* KO mice, respectively. **i** Immunoblots of PSD95 and synaptophysin of crude synaptosome fraction extracted from WT and *Pink1* KO cortical tissues. Actin is detected as a loading control. **j** Quantitative analysis of synaptic proteins for experiments shown in **i**. *n* = 8 mice per genotype. **k** Representative traces of mEPSCs recorded from somatosensory cortex layer 2/3 neurons of 8-week-old WT and *Pink1* KO mice. **l** Quantitation of mEPSCs frequencies and amplitudes for experiments shown in **k**. Eleven cells from three WT and 11 cells from five Pink1 KO mice were analyzed. *P < 0.05, **p < 0.01, ***p < 0.001, ****p < 0.0001. Student’s *t*-test

We next examined whether PINK1 regulates neuronal connections in intact brains in vivo. Golgi staining of cortical slices at postnatal day 14 (P14) showed that density of spines and mushroom/thin spines at secondary apical dendrites of layer 2/3 neurons in somatosensory cortex was reduced in *Pink1* KO mice (57.09 ± 3.210 total spines/100 µm; 51.81 ± 3.003 mushroom/thin spines/100 µm) comparing to that of their WT littermates (70.51 ± 3.152 total spines/100 µm; 65.39 ± 2.966 mushroom/thin spines/100 µm). By contrast, the density of filopodium was increased in *Pink1* KO neurons (5.96 ± 0.394 protrusions/100 µm) compared to that of WT neurons (2.48 ± 0.232 protrusions/100 µm). However, the density of stubby spines in neurons remained similar in *Pink1* KO and WT mice (Fig. 2a, b). Synapse density in hippocampal CA1 stratum radiatum and somatosensory cortex Layer 2/3, quantified by colocalization of pre- and post-synaptic puncta (Synaptophysin and PSD95), revealed a significant lower synapse density in both hippocampus and somatosensory cortex of *Pink* KO mice than that of WT littermates at age of 8 weeks (Fig. 2c, d). TEM analysis on a hippocampal or cortical slice of 8-week-old mice showed smaller postsynaptic density (PSD) in *Pink1* KO neurons than that of WT neurons (Fig. 2e, f). Notably, the presynaptic bouton in *Pink1* KO neurons was smaller and contained less synaptic vesicles compared to that in WT neurons (Fig. 2g, h, bouton size: 0.3625 ± 0.018 µm^2^ in WT neurons, 0.3038 ± 0.014 µm^2^ in *Pink1* KO neurons; number of synaptic vesicles per bouton: 52.74 ± 2.323 in WT neurons; 41.52 ± 1.731 in *Pink1* KO neurons). Results suggest defective presynaptic assembly in *Pink1* KO neurons. Consistently, immunoblot analysis of crude synaptosome (P2 fraction) extracted from cortical tissues showed that PSD95 was reduced in *Pink1* KO mice (Fig. 2i, j, Supplementary Fig. S2c, d) compared to that of their WT littermates. Synaptic transmission analysis showed that frequency and amplitude of miniature excitatory postsynaptic currents (mEPSCs) of somatosensory cortex Layer 2/3 and hippocampal CA1 neurons were significantly reduced in *Pink1* KO neurons than those of WT control neurons (Fig. 2k, l, Supplementary Fig. S3c, d). Paired-pulse facilitation (PPF) analysis of somatosensory cortex layer 2/3 neurons showed increased PPF in *Pink1* KO mice compared to that in WT mice, indicating a lower probability of releasing synaptic vesicles (Supplementary Fig. S3e, f). Results establish a physiological role of PINK1 in excitatory neuronal connectivity in hippocampal and cortical circuits.

### PINK1 is required for LTP induction and maintenance

**Fig. 3:**
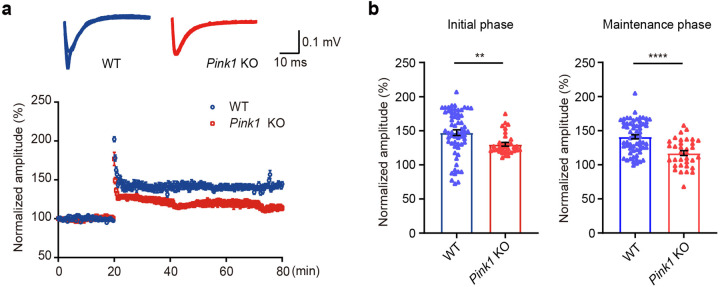
PINK1 is essential for LTP induction and maintenance. LTP was induced at the Schaffer collateral-CA1 synapses of acute hippocampal slices from 8-week-old WT (blue) and *Pink1* KO mice (red). Amplitudes of the fEPSPs evoked by TBS stimulation were normalized to the baseline (defined as 100%). Representative traces are fEPSPs at the basal line and 55 min after TBS (**a**). Quantitative analysis is shown (**b**). Note that PINK1 deficiency impaired TBS-induced LTP at Schaffer collateral-CA1 synapses in both initial (0–10 min post-TBS) and maintenance phase (40–60 min post-TBS). *n* = 63 and 34 recording for five WT and five *Pink1* KO mice, respectively. **p < 0.01, ****p < 0.0001. Student’s *t*-test

Next, we examined whether PINK1 regulates LTP induced by theta-burst stimulation (TBS) of the Schaffer collateral pathway in acute hippocampal slices. The field excitatory postsynaptic potentials (fEPSPs) recorded in *Pink1* KO Schaffer collateral-CA1 synapses was greatly reduced at both initial (0–10 min after TBS, *Pink1* KO 130.2% of baseline, WT 147.4% of baseline) and maintenance phase (40–60 min after TBS, *Pink1* KO 117.1% of baseline, WT 140.9% of baseline) post-TBS induction (Fig. 3). Therefore, PINK1 is required for not only induction but also maintenance of LTP.

### PINK1 regulates synapse maturation through Drp1^S616^ phosphorylation

**Fig. 4:**
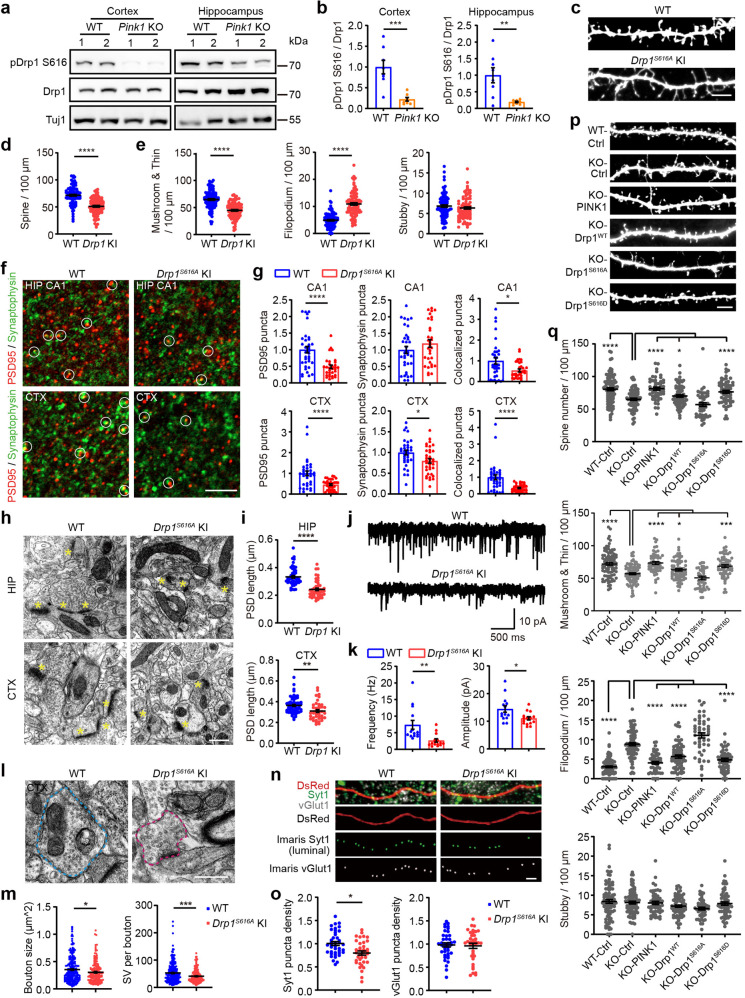
Phosphorylation of Drp1^S616^ mediates the PINK1 effect on synapse maturation. **a** Representative immunoblots of phosphorylated Drp1^S616^ (pDrp1 S616) of crude synaptosome fraction extracted from cortex and hippocampus of WT and *Pink1* KO tissues (P16.5). Total Drp1 (Drp1) and neuronal marker Tuj1 were immunodetected as controls. **b** Quantitation analysis of experiments presented in **a**. *n* = 8 mice/genotype. **P < 0.01, ***P < 0.001. Student’s *t*-test. **c** Representative images of dendritic spines of cortical neuronal cultures derived from WT or *Drp1*^*S616A*^ KI mice. Bar = 5 μm. **d**, **e** Quantification of protrusions for experiments presented in **c**. *n* = 93–98 dendrites/condition from three independent experiments. ****P < 0.0001. Student’s *t*-test. **f** Representative images of PSD95 (red) and synaptophysin (green) puncta in hippocampal CA1 stratum radiatum (CA1) or layer 2/3 of somatosensory cortex (CTX) of 8-week-old WT or *Drp1*^*S616A*^ KI mice. White circles indicate colocalized PSD95 and synaptophysin puncta. Bar = 5 μm. **g** Quantitation of synaptic puncta density for experiments presented in **f** using Imaris software. Five to six brain sections of each mouse from six WT and six *Drp1*^*S616A*^ KI mice were analyzed. *P < 0.05, ****P < 0.0001. Student’s *t*-test. **h** Representative TEM images of hippocampal CA1 (HIP) and somatosensory cortex (CTX) from WT and *Drp1*^*S616A*^ KI mice. Synapses are marked by yellow asterisks. Bar = 0.5 μm. **i** Quantitation of PSD length for experiments presented in **h**. HIP: *n* = 49 sections from five WT, *n* = 48 sections from five *Drp1*^*S616A*^ KI; CTX: *n* = 64 sections from five WT, *n* = 41 sections from five *Drp1*^*S616A*^ KI. **P < 0.01, ****P < 0.0001. Student’s *t*-test. **j** Representative traces of mEPSCs recorded from somatosensory cortex layer 2/3 neurons of 8-week-old *Drp1*^*S616A*^ KI mice and their WT littermates. **k** Quantitation of mEPSCs frequencies and amplitudes for experiments shown in **j**. *n* = 14 and 15 cells from five WT and five *Drp1*^*S616A*^ KI mice, respectively. *P < 0.05, **P < 0.01. Student’s *t*-test. **l** Representative TEM images of presynaptic boutons in the somatosensory cortex of WT and *Drp1*^*S616A*^ KI mice. Blue dash lines: presynaptic bouton with mitochondria. Magenta dash lines: presynaptic bouton without mitochondria. Bar = 0.5 μm. **m** Quantitation of bouton size and synaptic vesicles per bouton for experiments presented in **l**. *n* = 238 and 180 from three WT and four *Drp1*^*S616A*^ KI mice, respectively. *P < 0.05, ***P < 0.001. Student’s *t*-test. **n** Cortical neurons (DIV 14) derived from WT or *Drp1*^*S616A*^ KI mice were transfected with plasmid encoding DsRed followed by immunodetection of synaptotagmin 1(Syt1) and vGlut1 at DIV 18. Representative images of Syt1-positive puncta (green, Syt1), vGlut1-positive puncta (gray, vGlut1), and axon (red, DsRed) were shown. Imaris software processed Syt1 staining (green, Imaris Syt1) and Imaris software processed vGlut1 staining (white, Imaris vGlut1) on axons were shown. Bar = 2 μm. **o** Quantification of the density of Synaptotagomin 1 and vGlut1 for experiments presented in **n**. The puncta density represents counts of puncta divided by the length of axons (>50 μm). *n* = 40 (WT) and 36 (*Pink1* KO) axons collected from three independent experiments. All data were normalized with the mean value of WT axons of each experiment. *P < 0.05. Student’s *t*-test. **p** Cortical neurons derived from *Pink1* KO mice were co-transfected a plasmid encoding EGFP (Pink1 KO-Ctrl) with plasmids encoding either PINK1 (Pink1KO-PINK1), Drp1 (Pink1 KO-Drp1^WT^), Drp1 S616A mutant (Pink1 KO-Drp1^S616A^), or Drp1 S616D mutant (Pink1 KO-Drp1^S616D^). Neurons derived from WT mice transfected with EGFP were included as a control (WT-Ctrl). Representative images of dendritic spines were shown. Bar = 5 μm. **q** Quantification of the proportion of different protrusions for experiments presented in **p**. *n* > 48 dendrites from three independent experiments per condition. *P < 0.05, ***P < 0.001, ****P < 0.0001. Student’s *t*-test

We have recently demonstrated that PINK1 directly phosphorylates Drp1^S616^.^23^ Drp1 promotes synapse formation by affecting mitochondrial fission.^5,8^ To explore the mechanism of PINK1 in regulating synapse maturation and plasticity, we looked into PINK1 substrate Drp1^S616^ that control mitochondrial dynamics. Phosphor-Drp1^S616^ levels were greatly reduced in hippocampal and cortical tissues of *Pink1* KO mice compared to those in WT littermates, consistent with the notion of which PINK1 phosphorylates Drp1^S616^ in neurons (Fig. 4a, b).

We further generated a *Drp1*^*S616A*^ knockin mouse line that harbored a site-specific serine (S) to alanine (A) mutation at the site corresponding to the human *Drp1*^S616^ site (NM_012062.5) (Supplementary Fig. S4a, b). The homozygous *Drp1*^*S616A*^ knockin mutant mice (*Drp1*^*S616A*^ KI) were viable and fertile and showed no gross abnormalities. Immunoblotting verified that Drp1^S616A^ mutation abolished Drp1^S616^ phosphorylation without affecting total Drp1 protein (Supplementary Fig. S4c). Interestingly, the density of total dendritic protrusions including mushroom/thin spine was decreased (Fig. 4c–e, 64.79 ± 1.643 spines/100 µm in WT neurons; 44.82 ± 1.513 spines/100 µm in *Drp1*^*S616A*^ KI neurons), but not stubby spines (Fig. 4c, e, 6.81 ± 0.288 spines/100 µm in WT neurons; 6.34 ± 0.272 spines/100 µm in *Drp1*^*S616A*^ KI neurons), while the density of filopodium was increased (Fig. 4c, e, 4.876 ± 0.269 protrusions/100 µm in WT neurons; 10.91 ± 0.459 protrusions/100 µm in *Drp1*^*S616A*^ KI neurons) in neurons derived from *Drp1*^*S616A*^ KI at DIV 18, resembling spine maturation defects in *Pink1* KO neurons. Consistently, synaptic density quantified by colocalization of synaptophysin and PSD95 in hippocampal CA1 stratum radiatum and somatosensory cortex layer 2/3 was markedly decreased in *Drp1*^*S616A*^ KI mice compared to that of their WT littermates (Fig. 4f, g). TEM analysis revealed that the *Drp1*^*S616A*^ KI neuronal synapses in the hippocampus and somatosensory cortex contain shorter PSD than WT neuronal synapses do (Fig. 4h, i, hippocampus: 0.3331 ± 0.009621 µm in WT synapses; 0.2425 ± 0.008871 µm in *Drp1*^*S616A*^ KI synapses; cortex: 0.3643 ± 0.009441 µm in WT synapses; 0.3104 ± 0.01372 µm in *Drp1*^*S616A*^ KI synapses). Moreover, the frequency of mEPSCs was dramatically decreased in *Drp1*^*S616A*^ KI neurons (Fig. 4j, k, mean frequency 7.38 ± 1.339 Hz in WT neurons; 2.68 ± 0.492 Hz in *Drp1*^*S616A*^ KI neurons), which is consistent with a decrease in spine density. mEPSCs amplitude was reduced in *Drp1*^*S616A*^ KI neurons (Fig. 4j, k, mean amplitude 14.43 ± 1.184 pA in WT neurons; 11.06 ± 0.5901 pA in *Drp1*^*S616A*^ neurons), suggesting a possible change in postsynaptic receptors. Similarly, synaptic transmission in hippocampal CA1 neurons also diminished to some extent in *Drp1*^*S616A*^ KI mice (Supplementary Fig. S4d, e). Immunoblot analysis of cortical crude synaptosome showed that PSD95 was reduced in *Drp1*^*S616A*^ KI mice (Supplementary Fig. S4f, g) compared to that of their WT littermates. Thus, Drp1^S616^ phosphorylation promotes excitatory synapse maturation that resembles PINK1 activity.

Consistent with findings in *Pink1* KO mice, TEM analysis showed that presynaptic bouton size and synaptic vesicle number per bouton were decreased in brain slices from the somatosensory cortex of *Drp1*^*S616A*^ KI mice compared to those of WT mice (Fig. 4l, m). We further studied whether phosphor-Drp1^S616^ regulates presynaptic exocytosis. Exocytosed synaptic vesicles in cultured neurons were labeled by an antibody against the luminal domain of the synaptic vesicle protein synaptotagmin 1. Results showed that the density of recycled synaptotagmin 1 puncta along axons was decreased in *Drp1*^*S616A*^ KI neurons (Fig. 4n, o), while the co-immunostained vGlut1 puncta density was comparable in *Drp1*^*S616A*^ KI and WT neurons (Fig. 4n, o). Furthermore, PPF was increased in *Drp1*^*S616A*^ KI mice compared with WT mice (Supplementary Fig. S3e, f). Results suggest that Drp1^S616^ phosphorylation is required for the establishment of functional presynaptic terminals that are undergoing presynaptic exocytosis.

We next determined whether PINK1 regulates synapse maturation via Drp1^S616^ phosphorylation. *Pink1* KO neurons were co-expressing EGFP with either Drp1^S616D^ (a phosphor-mimetic variant of Drp1), Drp1^S616A^ (a phosphor-null variant of Drp1), or Drp1^WT^. As a control, neurons derived from WT control mice were co-transfected with empty control plasmid and EGFP plasmid. The numbers and morphology of dendritic spines in each group were quantified at DIV 18. *Pink1* KO neurons expressing Drp1^S616D^ or Drp1^WT^, but not Drp1^S616A^, resulted in a significant increase of density of total spines (Fig. 4p, q, 70.16 ± 1.918 spines/100 µm in *Pink1* KO neurons expressing Drp1^WT^; 56.93 ± 2.919 spines/100 µm in *Pink1* KO neurons expressing Drp1 ^S616A^ and 76.49 ± 2.322 spines/100 µm in *Pink1* KO neurons expressing Drp1^S616D^) and mushroom/thin spines (Fig. 4p, q, 62.97 ± 1.854 spines/100 µm in *Pink1* KO neurons overexpressed Drp1^WT^; 50.53 ± 2.787 spines/100 µm in *Pink1* KO neurons overexpressed Drp1 ^S616A^ and 68.62 ± 2.161 spines/100 µm in *Pink1* KO neurons overexpressed Drp1^S616D^) comparing to *Pink1* KO neurons transfected with control empty plasmid (Fig. 4p, q, 65.07 ± 1.702 total spines /100 µm; 56.97 ± 1.608 mushroom/thin spines/100 µm). In contrast, filopodium density was decreased in *Pink1* KO neurons expressing Drp1^S616D^ or Drp1^WT^, but not the neurons expressing Drp1^S616A^ (Fig. 4p, q, 5.674 ± 0.393 protrusions/100 µm in *Pink1* KO neurons expressing Drp1^WT^; 11.09 ± 0.665 protrusions/100 µm in *Pink1* KO neurons expressing Drp1 ^S616A^; and 4.832 ± 0.407 protrusions/100 µm in *Pink1* KO neurons expressing Drp1^S616D^) comparing to that in *Pink1* KO neurons transfected with control empty plasmid (Fig. 4p, q, 8.84 ± 0.387 protrusions/100 µm). Notably, density of total spines and each type of dendritic protrusions in *Pink1* KO neurons expressing Drp1^S616D^ (Fig. 4p, q, 76.49 ± 2.322 total spines/100 µm; 68.62 ± 2.161 mushroom/thin spines/100 µm; 4.83 ± 0.407 filopodium/100 µm) were comparable to those in neurons derived from wild-type mice (Fig. 4p, q, 80.41 ± 2.258 total spines/100 µm; 72.00 ± 2.098 mushroom/thin spines/100 µm; 3.05 ± 0.222 filopodium/100 µm). Thus, Drp1^S616D^ expression is sufficient to restore spine development defects of *Pink1* KO neurons. Therefore, Drp1^S616^ phosphorylation is downstream of PINK1 in regulating spine maturation.

### PINK1-dependent Drp1^S616^ phosphorylation is critical for LTP induction and maintenance

**Fig. 5:**
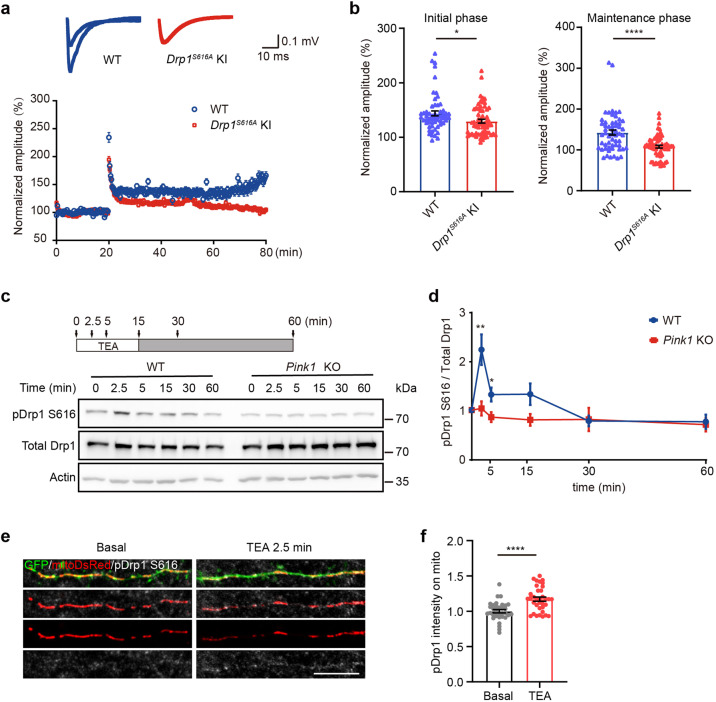
PINK1-mediated Drp1^S616^ phosphorylation is required for LTP induction. **a** LTP was induced at the Schaffer collateral-CA1 synapses of acute hippocampal slices from 2-month-old WT (blue) and *Drp1*^*S616A*^ KI (red) mice. Amplitudes of the fEPSPs evoked by TBS were normalized to the baseline (defined as 100%). Representative traces are fEPSPs at the basal line and 55 min after TBS. **b** Quantitation of induced fEPSPs at initial (0–10 min after TBS) and maintenance phase (40–60 min after TBS) of WT (blue) and *Drp1*^*S616A*^ KI (red) mice. *n* = 57 and 63 recordings of 5–6 WT and *Drp1*^*S616A*^ KI mice, respectively. *P < 0.05, ****P < 0.0001. Student’s *t*-test. **c** Cortical neurons (DIV 18) derived from WT or *Pink1* KO mice were treated with 25 mM TEA for 2.5, 5, and 15 min (arrows, open bar, upper panel). After 15 min treatment, TEA was washed, neurons were harvested at the indicated time (arrows, filled bar, upper panel). Levels of phosphor-Drp1^S616^ (pDrp1 S616), total Drp1, and actin were shown (lower panel). **d** Quantitative analysis of phosphor-Drp1^S616^ normalized with total Drp1 for experiments presented in **c**. WT: blue. *Pink1* KO: red. *n* = 7–12 treatment. *P < 0.05, **P < 0.01. Multiple *t*-test. **e** Cortical neurons (DIV 10) derived from WT mice were expressing mitoDsRed (red) and GFP (green). At DIV 18, neurons were treated with either solvent (Basal) or 25 mM TEA for 2.5 min, fixed, and stained with anti-pDrp1^S616^ antibody (gray). Representative images of pDrp1^S616^ and mitochondria were shown. Bar = 10 μm. **f** Quantitation of pDrp1 S616 intensity on mitochondria. Mitochondria were reconstructed by Imaris. The mean intensity of pDrp1S616 on mitochondria was calculated. Data were normalized with the mean value of pDrp1^S616^ intensity of the Basal group in the experiment. *n* = 34 and 35 dendrites from Basal and TEA group of three independent experiments. ****P < 0.0001. Student’s *t*-test

To further explore the physiological role of Drp1^S616^ phosphorylation in LTP, we induced LTP in Schafer collateral pathway at acute hippocampal slices prepared from *Drp1*^*S616A*^ KI mice and their WT control littermates by TBS stimulation. Consistent with findings from *Pink1* KO mice, fEPSPs recorded at CA1 were attenuated in neurons of *Drp1*^*S616A*^ KI mice compared to that in neurons of WT control mice at both initiation (0–10 min after TBS, *Drp1*^*S616A*^ KI 129.5 ± 3.479% of baseline; WT 143.8 ± 4.547% of baseline) and maintenance phase (40–60 min after TBS, *Drp1*^*S616A*^ KI 108 ± 3.495% of baseline; WT 142.7 ± 6.189% of baseline) of LTP (Fig. 5a, b). Thus, *Drp1*^*S616*^ phosphorylation plays an essential role in LTP induction and maintenance.

We next examined Drp1^S616^ phosphorylation following LTP-induction by employing a tetraethylammonium chloride (TEA)-induced protocol in primary cultured neurons.^27–29^ The total Drp1 remained unchanged at all time points examined after 25 mM TEA treatment for 15 min (Fig. 5c, d). Notably, a rapid increase of Drp1^S616^ phosphorylation peaked at 2.5 min post TEA treatment (Fig. 5c, d, 216.1 ± 29.4% of baseline, *p* < 0.01). Phosphor-Drp1^S616^ remains increase at 15 min after TEA administration, although not reaching statistical significance (Fig. 5c, d, 130.6 ± 20.7% of baseline, *p* = 0.073). In contrast, TEA did not induce Drp1^S616^ phosphorylation in *Pink1* KO neurons (Fig. 5c, d). Moreover, phosphorylated Drp1^S616^ was accumulated on mitochondria after cLTP treatment, which is consistent with a previous report that Drp1^S616^ is required for cLTP fission burst^6^ (Fig. 5e, f). Results suggest that PINK1 phosphorylates Drp1^S616^ upon LTP induction, thereby contributing to LTP induction and maintenance.

### PINK1-mediated Drp1^S616^ phosphorylation regulates mitochondrial fission during synapse development

**Fig. 6:**
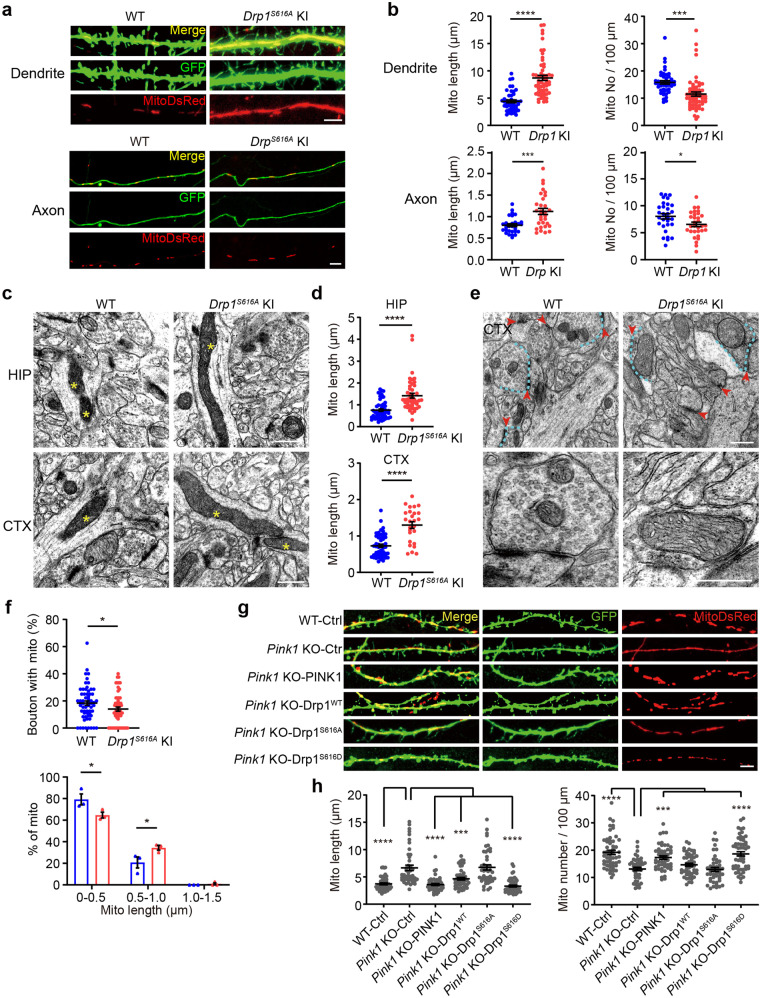
PINK1-mediated Drp1^S616^ phosphorylation regulates mitochondrial dynamics during synapse maturation. **a** Cortical neurons (DIV 6-8) derived from WT and *Drp1*^*S616A*^ KI mice were transfected with plasmids encoding MitoDsRed (red) and EGFP (green) followed by fixation at DIV 18. Merged images are shown in the top panels. Bar = 5 μm. **b** Quantitation of dendritic and axonal mitochondrial length (Mito length) and number (Mito No) for experiments present in **a**. *n* = 45–58 dendrites/genotype and 30 axons/genotype from three independent experiments per condition. *P < 0.05, ***p < 0.001, ****p < 0.0001. Student’s *t*-test. **c** Representative TEM images of dendritic mitochondria (yellow asterisks) from hippocampal CA1 (HIP) and somatosensory cortex (CTX) of 8-week-old WT and *Drp1*^*S616A*^ KI mice. Bar = 0.5 μm. **d** Quantitation analysis of dendritic mitochondrial length for experiments presented in **c**. HIP: *n* = 56 and 47 sections from three to four WT and *Drp1*^*S616A*^ KI mice, respectively*;* CTX: *n* = 51 and 26 sections from three to four WT and *Drp1*^*S616A*^ KI, respectively. ****P < 0.0001. Student’s *t*-test. **e** Representative TEM images of synapses and presynaptic mitochondria in somatosensory cortex sections of 8-week-old WT and *Drp1*^*S616A*^ KI mice. Blue dash lines: presynaptic bouton with mitochondria. Red arrows: synapse with postsynaptic density structure (upper panels). Amplified images were also shown (lower panels). Bar = 0.5 μm. **f** Quantitative analysis of presynaptic bouton with mitochondria (Bouton with mito) and presynaptic mitochondrial sizes (% of mito). Presynaptic bouton with mitochondria was analyzed for 62 sections from three WT and 59 sections from three *Drp1*^*S616A*^ KI mice. The mitochondrial size was done with 80 mitochondria for three WT and 72 mitochondria for three *Drp1*^*S616A*^ KI mice. *P < 0.05. Student’s *t*-test. **g** Cortical neurons derived from *Pink1* KO mice were co-transfected with plasmids encoding MitoDsRed (Red), EGFP (Green), and either empty plasmid (Pink1 KO-Ctrl), PINK1 (Pink1 KO-PINK1), Drp1 (Pink1 KO-Drp1^WT^), Drp1 S616A mutant (Pink1 KO-Drp1^S616A^), or Drp1 S616D mutant (Pink1 KO-Drp1^S616D^) at DIV 6-8, followed by imaging at DIV 18. Neurons derived from WT mice co-transfected with plasmids encoding MitoDsRed and EGFP were included as a control (WT-Ctrl). Representative images of dendritic spines and corresponding mitochondria in transfected neurons are shown. Bar = 5 μm. **h** Quantitation analysis of dendritic mitochondrial length (Mito length) and dendritic mitochondrial number for experiments presented in **g**. *n* = 45–55 dendrites/condition from three independent experiments. ***P < 0.001, ****P < 0.0001. One-way ANOVA followed by Dunnett’s multiple comparison test

We next examined whether PINK1 regulates synapse morphogenesis and maturation via promoting Drp1^S616^ phosphorylation-mediated mitochondrial fission in neurons. Cortical neuronal cultures derived from *Drp1*^*S616A*^ KI and their WT control littermates were co-expressed with MitoDsRed (to label mitochondria) and EGFP (to visualize neuronal morphology). *Drp1*^*S616A*^ KI neurons displayed fewer but enlarged dendritic and axonal mitochondria at DIV 18 compared to those of WT control neurons, which is consistent with the observation in *Pink1* KO neurons (Fig. 6a, b). TEM analysis showed that dendritic mitochondrial size is larger in *Drp1*^*S616A*^ KI hippocampal and cortical neurons than that in WT control neurons at the age of 8 weeks (Fig. 6c, d). In addition, presynaptic boutons in *Drp1*^*S616A*^ KI neurons were less frequent to contain mitochondria, although the large-sized mitochondria (0.5–1 µm in length) were observed within presynaptic boutons of *Drp1*^*S616A*^ KI neurons (Fig. 6e, f). Thus, Drp1^S616^ phosphorylation plays vital role in mitochondrial fission and distribution in neurons.

Next, we examined whether Drp1^S616^ phosphorylation is involved in PINK1-mediated mitochondrial fission. Primary neuronal cultures derived from *Pink1* KO mice at DIV 6–8 were co-expressed MitoDsRed and EGFP with either Drp1^S616D^, Drp1^S616A^, or Drp1^WT^. Expression of Drp1^S616D^, but not Drp1^S616A^, restored dendritic mitochondrial fission abnormality in *Pink1* KO neurons (Fig. 6g, h). Expression of Drp1^S616D^ resulted in a reduced length (3.30 ± 0.169 µm) and an increased density of mitochondria (18.66 ± 0.8014 mitochondria/100 µm) in *Pink1* KO neurons compared to that of control neurons (6.652 ± 0.492 µm in length, 13.11 ± 0.617 mitochondria/100 µm). Expression of Drp1^WT^ partially rescued mitochondria fission deficiency in *Pink1* KO neurons (Fig. 6g, h). It is likely that other kinases for Drp1^S616^ exist as shown in *Drosophila*.^23^ Together, results indicate that phosphor-Drp1^S616^ mediates PINK1 effects on dendritic mitochondrial fission during synapse development.

To test whether PINK1 affects local ATP homeostasis in dendrites, PercevalHR, a genetically encoded fluorescent ATP sensor,^30^ was expressed in cultured *Pink1* KO and WT cortical neurons. The fluorescence intensity ratio of F488nm/F405nm, which indicates the intracellular ATP/ADP ratio, was significantly lower in *Pink1* KO dendrites than that in WT dendrites (Supplementary Fig. S5a, b). Results suggest that defects of mitochondria fission in *Pink1* KO neurons reduces dendritic energy supply. Meanwhile, cortical neurons (DIV 18) derived from *Pink1* KO mice treated with 1 mM piracetam, a chemical to improve mitochondrial function and increase ATP production,^31^ partially rescued spine defects of *Pink1* KO neurons (Supplementary Fig. S6a, b). Thus, PINK1/phosphor-Drp1^S616^ regulates mitochondrial dynamics and ATP production to modulate spine maturation.

### PINK1-mediated Drp1^S616^ phosphorylation regulates synaptic localization and invasion of mitochondria

**Fig. 7:**
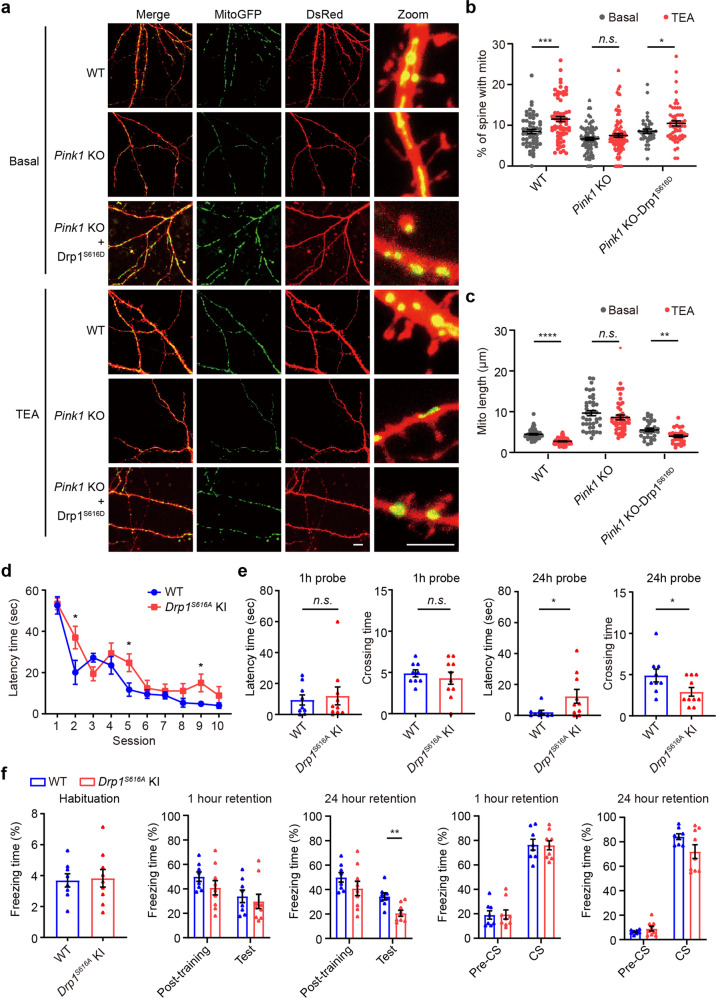
Phosphor-Drp1^S616^ regulates mitochondrial redistribution in dendrites and spatial and fear memory. **a** Cortical neurons (DIV 6–8) derived from WT or *Pink1* KO mice were co-transfected with plasmids encoding MitoGFP (Green) and DsRed (red), along with a plasmid encoding Drp1 S616D mutant (Drp1^S616D^). Representative images of dendrites with mitochondria in transfected neurons were shown. Amplified images of each transfection were included (Zoom). Bar = 10 μm. **b** Quantitation of dendritic spines with mitochondria in each condition for experiments presented in **a**. *n* = 31–96 dendrites/condition from three independent experiments. *p < 0.05, ***p < 0.001, n.s. not significant. Student’s *t-*test. **c** Quantitative analysis of mitochondrial length (Mito length) in dendrites from experiments shown in **a**. *n* = 30–54 dendrites/condition from three independent experiments. **p < 0.01, ****p < 0.0001, n.s. not significant. Student’s *t*-test. **d** Spatial learning curves during Morris water maze (MWM) training of WT (blue) and *Drp1*^*S616A*^ KI mice (red). *n* = 9–10 mice/genotype. **e** Quantification of latency time and crossing time for 1-h-probe and 24-h-probe trials. *n* = 9–10 mice/genotype. **f** Freezing time of WT (blue bar) and *Drp1*^*S616A*^ KI (red bar) mice in the contextual or cued fear memory test. *n* = 8–9 mice/genotype. *p < 0.05, **p < 0.01. Student’s *t*-test

At DIV 18, around 8% of dendritic protrusions contained mitochondria in wild-type control neurons. However, dendritic protrusions with mitochondria were reduced to around 6% in *Pink1* KO cortical neurons (Fig. 7a, b, basal level). Notably, this reduction of mitochondria in dendritic protrusions was rescued by expressing Drp1^S616D^. Results suggest that PINK1 increases mitochondrial incursion into dendritic protrusions to promote synapse/spine maturation via promoting Drp1^S616^ phosphorylation-mediated mitochondrial fission.

We next test whether synaptic redistribution of mitochondria occurs upon LTP induction via the PINK1/phosphor-Drp1^S616^ pathway. Cortical neurons (DIV 18) labeled by DsRed and MitoGFP were treated with TEA (25 mM) for 15 min followed by fixing neurons 30 min posttreatment. Compared to the basal level, TEA treatment resulted in a ~35% increase of dendritic protrusions containing mitochondria (Fig. 7a, b, 8.48 ± 0.531% in basal level, 11.52 ± 0.645% in TEA treatment). In contrast, TEA treatment elicited little change of dendritic protrusions containing mitochondria in *Pink1* KO neurons (6.662 ± 0.3822% in basal level, 7.477 ± 0.450% in TEA treatment). Expression of Drp1^S616D^ restored mitochondrial invasion into protrusions in *Pink1* KO neurons (8.91 ± 0.642% in basal level, 11.06 ± 0.712% in TEA treatment). Moreover, TEA-induced mitochondrial fragmentation was abolished in *Pink1* KO neurons that was rescued by introducing Drp1^S616D^ (Fig. 7c). Results suggest that PINK1-mediated Drp1^S616^ phosphorylation regulates TEA-induced mitochondrial incursion into synaptic compartments.

### *Drp1*^*S616A*^ KI mice exhibited impaired hippocampal-dependent memory

The appearance and gross motor behavior of *Drp1*^*S616A*^ *KI* mice were normal at age of 5 months (data not shown). We next explored the roles of phosphor-Drp1^S616^ in learning and memory by employing Morris water maze (MWM) task tests. 7-month-old mice were trained for swimming tests for 2 sessions/day for 5 days. In the first session, *Drp1*^*S616A*^ KI mice and their wild-type control littermates spend similar time to reach the platform, indicating no difference in locomotion activity and swim capacity between genotypes (Fig. 7d). Wild-type control mice learned rapidly accompanying training sessions and spent significantly less time to find a platform than *Drp1*^*S616A*^ KI mice did in sessions 2, 5, and 9 (Fig. 7d). To test spatial memory, probe trials were performed at 1 and 24 h after the final training session in the same swimming arena with the removed platform. In the 24-h probe tests, *Drp1*^*S616A*^ KI mice spent significantly more time to the previous platform location (Fig. 7e, 2.1 ± 1.198 s in WT mice and 12.4 ± 4.474 s in *Drp1*^*S616A*^ KI mice) and make fewer crossings in the quadrant than that of their WT control littermates (Fig. 7e, 4.9 ± 0.772 times in WT mice, 2.9 ± 0.526 times in *Drp1*^*S616A*^ KI mice). Results suggest that long-term memory retention of *Drp1*^*S616A*^ KI mice was impaired. In contrast, latency time and crossing time in the target area were comparable in WT mice and *Drp1*^*S616A*^ KI mice in the 1-h probe tests (Fig. 7e). Results indicate that Drp1^S616^ phosphorylation is pivotal for hippocampal-dependent spatial learning and memory.

To determine the roles of phosphor-Drp1^S616^ in fear memory, the contextual and the cued fear conditioning tests were performed. To assess the short-term and long-term contextual memory in the fear conditioning task, 9-month-old mice were tested in the training context at 1- or 24-h after pairing of conditioned (tone) and unconditioned (electrical footshock) stimuli. *Drp1*^*S616A*^ KI mice froze less often than WT littermates did at 24-h post-training (Fig. 7f, 34.33 ± 2.927% in WT mice and 20.62 ± 2.39% in *Drp1*^*S616A*^ KI mice), whereas *Drp1*^*S616A*^ KI mice had less freezing time compared to WT littermates at 1-hour post-training. However, it did not reach a statistical significance (Fig. 7f, 33.85 ± 5.086% in WT mice and 29.7 ± 5.823% in *Drp1*^*S616A*^ KI mice). Results suggest that *Drp1*^*S616A*^ KI mice had a more robust effect on long-term contextual memory. In contrast, assessment of auditory fear memory revealed that the freezing time in novel context during the tone-period tested at both 1- or 24-h after training was comparable between two genotypes (Fig. 7f), suggesting the cued fear memory remained intact in *Drp1*^*S616A*^ KI mice. We noted that *Drp1*^*S616A*^ KI and the wild-type control mice had comparable freezing levels in the habituation period before associated training or the period immediately after training (Fig. 7f). No genotype differences in baseline anxiety, locomotor activity, fear response, and associative fear learning. Thus, phosphor-Drp1^S616^ is required preferentially for the consolidation of contextual fear memory. Drp1^S616^ phosphorylation plays an important role in hippocampal-dependent spatial and fear memory.

## Discussion

Mitochondrial plasticity underlies synapse formation and functions. Dendritic mitochondrial dynamics are involved in the induction of NMDA receptor-dependent LTP, stabilization of long-term plasticity, and plasticity-induced local protein translation.^6,7,32^ Axonal mitochondria dynamics are required for presynaptic release and short-term plasticity.^33,34^ However, molecular mechanisms of how mitochondrial dynamics modulates synapse formation and function are barely understood.

In this study, we demonstrate that PINK1-mediated Drp1^S616^ phosphorylation regulates mitochondrial dynamics to modulate the development and maturation of excitatory circuits and synaptic plasticity in the hippocampus and cortex. Three lines of evidence support this notion. First, PINK1 phosphorylates Drp1^S616^ to promote mitochondrial fission, increase mitochondrial density and mitochondria in dendritic protrusion and presynaptic bouton. Second, cLTP induction elevates PINK1-mediated Drp1^S616^ phosphorylation and promotes dendritic mitochondrial fission. LTP induction and maintenance are suppressed in *Pink1* KO or *Drp1*^*S616A*^ KI mice. Modulation of mitochondrial morphology likely allows its localization and energy supply to dendritic protrusions. Finally, the contextual fear memory and spatial memory are impaired in *Drp1*^*S616A*^ KI mice, highlighting an essential role of Drp1^S616^ phosphorylation in learning and memory.

PINK1/parkin are well known to regulate mitophagy.^35^ PINK1 also regulates mitochondrial dynamics. We have recently shown that PINK1 directly phosphorylates Drp1^S616^, therefore, controls mitochondrial fission.^23^ Consistently, overexpression of PINK1 increases mitochondrial fission in primary rat hippocampal neurons at DIV12.^22^ In *Drosophila*, PINK1/Parkin is shown to regulate mitochondrial morphological dynamics.^17–20^ This study suggests that PINK1 regulates mitochondrial fission via phosphorylating Drp1^S616^, resulting in modulation of maturation of synaptic circuit and synaptic plasticity for memory formation and consolidation. One potential mechanism supported by this study is that PINK1/phosphor-Drp1^S616^-promoted dendritic mitochondrial fission to generate smaller mitochondria to trafficking to pre- and postsynaptic terminals to provide the required energy. The majority of the energy in neurons is consumed at synapses to support synapse development and synaptic functions, such as synaptic transmission and plasticity.^36^ Mitochondria are recognized as the main powerhouse to fuel synaptic events by producing ATP. Dendritic mitochondria are shown to provide ATP to support synaptic protein translation during synaptic plasticity^32^ and to generate ROS to maintain the long-term phase of structural LTP.^7^ In contrast to this study, silencing PINK1 in primary mouse hippocampal neurons at DIV 7, the time point for active synaptogenesis prior to dendritic spines maturation,^22,37^ is reported to cause mitochondrial fragmentation.^38^ Furthermore, CDK5-mediated Drp1^S616^ phosphorylation leads to fission inhibition in young neuronal cultures at DIV 10.^39^ CDK5 phosphorylates Drp1 in multiple serines, including S585 and S579, with different functional consequences. One explanation is that PINK1 regulates mitochondrial dynamics in a context-dependent or development-dependent manner. In this study, Drp1^S616^ phosphorylation induced by cLTP is abolished in *Pink1*-deficient neurons, suggesting a PINK1-dependent mechanism.

This study also reveals a molecular mechanism of mitochondria in regulating maturation and experience-dependent refinement of excitatory neuronal networks. The electrophysical field recording to assess TBS-induced LTP in a hippocampal acute slice from *Drp1*^*S616A*^ KI mice suggests an important role of Drp1^S616^ phosphorylation in LTP induction in hippocampal CA3-CA1 synapses. Furthermore, a PINK1-dependent elevation of Drp1^S616^ phosphorylation follows TEA-induced LTP. Consistently, NMDAR-dependent LTP induction prompted a rapid burst of Drp1 dependent-dendritic mitochondrial fission.^6^ Glycine-induced cLTP stimulates a burst of dendritic mitochondrial fission events that is abolished by expressing Drp1^S616A^ in cultured neurons.^6^ This study establishes a direct link between PINK1-mediated Drp1^S616^ phosphorylation and LTP induction.

Mutations in PINK1 are linked to Parkinson’s disease, a neurodegenerative movement disease.^40^ Results from this study establish a functional link of PINK1 and synapses. In skin fibroblast-derived from PD patients harboring PINK1 mutation, phosphor-Drp1^S616^ is lower than that in skin fibroblasts derived from control individuals.^23^ The mechanism may contribute to the pathogenesis of the disease.

**Fig. 8:**
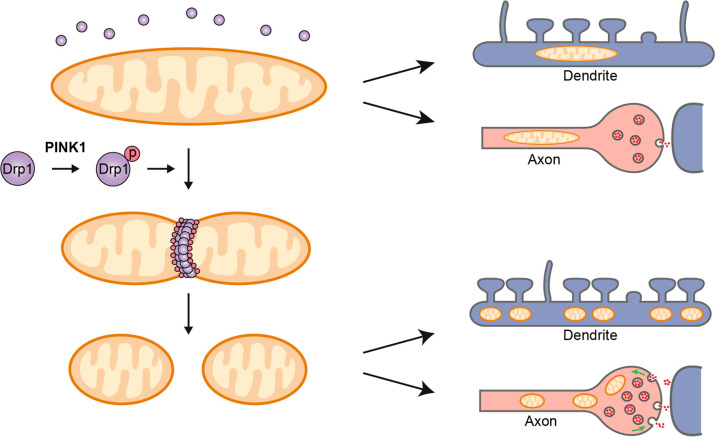
Schematic illustration of PINK1-mediated Drp1^S616^ phosphorylation to regulate synaptic transmission and plasticity via mitochondrial fission. Fused mitochondria (left top panel) become fissed (left bottom panel) via a PINK1/phosphor-Drp1^S616^ mediated mechanism (left middle panel). Fused mitochondria are difficult to traffic to presynaptic terminals and dendrites (right upper panel) compared to fissed mitochondria (right lower panel)

In summary, this study provides to our knowledge the first in vivo evidence of functional regulation of Drp1 by phosphorylation and demonstrates mitochondrial fission dynamics and distribution through coordination of PINK1 kinase activity and Drp1^S616^ phosphorylation as a mechanism essential for synapse maturation, neuronal connectivity, and synaptic plasticity (illustrated in Fig. 8), providing a new avenue to study roles of mitochondria in establishment and refinement of the neuronal circuit and learning and memory.

## Materials and methods

### Mice

*Pink1* KO mice were previously described.^41^ Methods for design of guide RNA, donor oligo, and production of *Drp1*^*S616A*^ knockin mice were described.^42^ Briefly, the guide RNA target sequence (ATGCCAGCAAGTCCACAGAA) was selected according to the on- and off-target scores from the CRISPR design web tool (http://www.benchling.com) and purchased from IDT (Coralville, Iowa, USA) in the two-part crRNA and tracrRNA format. To form a guide RNA duplex, we mixed crRNA and tracrRNA in 100 µl nuclease-free duplex buffer (IDT) at a final concentration of 1 mM each. The mix was heated at 95 °C for 5 min and allowed to cool on the benchtop to room temperature. To prepare the injection mixture, we incubated guide RNA duplex and Cas9 protein (IDT) at 37 °C for 10 min to form ribonucleoproteins and added the single-stranded DNA donor oligos (IDT) with S596A mutation (S596 of mouse Drp1 variant 3 (NM_001276340.1) corresponds to human Drp1 residue S616 (NM_012062.5). The final concentrations were 100 ng/µl guide RNA duplex, 200 ng/µl Cas9 protein, 50 ng/µl S596A donor oligo. The mutant mice were generated by injection of the mixture into the cytoplasm of fertilized eggs of C57BL/6 genetic background, using a piezo-driven microinjection technique.^43^ Injected eggs were transferred into the oviductal ampulla of pseudopregnant CD-1 females on the same day. Pups were born and genotyped by PCR using the primers (5′-TGTGTTTTCAGGTCCCATCTGC, and 5′-CCTCACAATCTCGCTGTTCTCG). The knockin allele was identified by SacII enzyme digestion and further confirmed by Sanger sequencing. Animals were housed in a controlled environment with a 12-h light/12-h dark cycle, with free access to water and a standard chow diet. Behavioral experiments were conducted between 10:00 a.m. and 5:00 p.m.

All animal procedures were carried out in accordance with either the Institutional Animal Care and Use Committee-approved protocol of Cincinnati Children’s Hospital or Medical Center or approved by the Ethics Review Committee for Animal Experimentation of Central South University.

### Primary neuronal cultures

Cortical or hippocampal neuronal cultures were prepared from embryonic day 16.5 mice and plated on poly-d-lysine coated coverslips as previously described.^44^ Briefly, hippocampi and cerebral cortices were isolated in cold HBSS and incubated with 0.025% trypsin at 37 °C for 20 min with gentle shaking every 5 min. After digestion, cells were dissociated with glass Pasteur pipettes ten times gently, filtered through 70 µm nylon cell strainer, and counted. Cells were plated at 4 × 10^5^ cells/well for high density and 2 × 10^5^ cells/well for low density in 12-well plates. Cultured cells were maintained in a neurobasal medium supplemented with 2% B27 and 2 mM glutamax at 37 °C and 5% CO_2_.

### Electrophysiology

Transverse brain slices (300 µm) were prepared from 6–8-week-old male mice as described previously.^45^ Slices were transferred to a holding chamber that contains artificial cerebrospinal fluid (124 mM NaCl, 3 mM KCl, 26 mM NaHCO_3_, 1.25 mM NaH_2_PO_4_, 1.2 mM MgCl_2_, 10 mM glucose, 2 mM CaCl_2_, pH 7.4, 305 mOsm, bubbled with 95% O_2_/5% CO_2_). The slices were allowed to recover at 31.5 °C for 30 min and then at room temperature for 1 h. Acute slices were transferred to a recording chamber continuously, which was perfused with oxygenated artificial cerebrospinal fluid (2 ml/min) at room temperature. For whole-cell patch-clamp recordings from the cortical layer 2/3 pyramidal cells, brain slices were visualized via input resistance-DIC by using an Axioskop 2FS equipped with Hamamatsu C2400-07E optics (Hamamatsu City, Japan). Basic electrophysiological properties were recorded when stable recordings were achieved with good access resistance (20 MΩ). The mEPSCs were recorded using an internal solution containing (in mM) 140 potassium gluconate, 10 HEPES, 0.2 EGTA, 2 Mg^2+^ATP, 2 NaCl, and 0.3 NaGTP and an external solution containing 10 µM bicuculline and 1 µM tetrodotoxin (TTX). The data were collected at 10 kHz and filtered with a low-pass filter at 2 kHz. Miniature events were analyzed in Clampfit 10.2 software (Molecular Devices, Sunnyvale, CA, USA) based on the waveforms of the events.

mEPSC of hippocampal CA1 neurons was recorded using patch pipettes with an impedance of 4–7 MΩ filled with the internal solution containing 120 mM CeCl, 10 mM HEPES, 10 mM TEA-Cl, 1 mM EGTA, 2 mM QX314-Cl, 4 mM Mg-ATP, 0.3 mM Na3GTP, 0.3 mM Phosphocreatine sodium, 0.4% biocytin, 285-295 mOsm, pH 7.2–7.25. The external solution contained 50 µM picrotoxin (PTX) and 1 µM tetrodotoxin (TTX). Voltage clamp recordings were achieved using a Multiclamp 700B amplifier (Molecular Devices, Sunnyvale, CA, USA). We used Axon Digidata 1550B with pclamp (V10.6) for data acquisition. Voltage and current signals were filtered at 2.4 kHz, respectively, and sampled at 50 kHz. Miniature events were analyzed in Clampfit 11.2 software (Molecular Devices, Sunnyvale, CA, USA) and MiniAnal software based on the waveforms of the events.^46^

PPF analysis, somatosensory cortex Layer 2/3 neurons were patched in internal solution (140 mM Cs-gluconate, 1 mM NaCl, 5 mM EGTA, 1 mM MgCl2, 1 mM CaCl2, 3 mM KOH, and 2 mM Mg-ATP and was titrated to a final pH of 7.2–7.3 with CsOH and an osmolality of 290–300 mOsm). Presynaptic and postsynaptic compartments were simultaneously clamped at −70 mV. Brief depolarization (two times of threshold, 0.1 ms) of presynaptic neuron elicited an AP, two 0.1-ms depolarizing step pulses were given 20 ms apart, with a 20 s interval between paired pulses. Postsynaptic currents (PSCs) were recorded. The peak amplitude of the response to the second pulse was averaged over ten trials and divided by the averaged peak amplitude of the response to the first pulse to give a paired-pulse ratio (P2/P1). Data were analyzed in Clampfit 11.2 software.^47^

For LTP induction, hippocampal slices (300 µm) were prepared as described above. For field potential recordings, acute hippocampal slices were placed on a Med64-multielectrode array (Alpha MED Scientific, Osaka, Japan). Field excitatory postsynaptic potentials (fEPSPs) were elicited and recorded via planar electrodes of the Probe AL-MED-P515A by aligning the electrodes and the stratum radiatum region of hippocampal slices. An input-output curve analysis was performed at the beginning of each recording to determine the appropriate stimulation intensity. Test stimuli at 30–40% of maximal intensity were delivered at 0.05 Hz and a stable baseline of fEPSPs of 20 min was established before LTP induction. The amplitude of peak fEPSPs was measured. LTP was induced using a theta-burst protocol comprised of three trains delivered every 10 s, each train containing ten bursts at 5 Hz, each burst containing four pulses at 100 Hz. LTP was induced at 10 µA above test intensity to ensure robust LTP induction. Recordings lasted for an hour after induction. Recordings and analysis were performed using Med64 Mobius Software (Alpha MED Scientific).

### Plasmids and transfection

Plasmid encoding C-terminal FLAG-tagged Pink1 was described previously.^41^ cDNA encoding *Drp1* (Human isoform 1) was cloned into pcDNA3.1/myc-his(B + ) (Invitrogen, San Diego, USA). Constructs encoding Drp1^S616A^ and Drp1^S616D^ mutants were generated using a QuickChange site-directed Mutagenesis Kit from Stratagene (La Jolla, USA). For most experiments, cells were transfected using calcium phosphate or Lipofectamine2000 (Invitrogen, Carlsbad, USA).

### Immunofluorescence and image analysis

Neurons were fixed with 4% (wt/vol) paraformaldehyde/4% (wt/vol) sucrose for 10 min at room temperature, permeabilized with 0.1% Triton X-100 in PBS for 15 min at room temperature, blocked with 5% (vol/vol) BSA in PBS for 1 h at room temperature, and incubated with the primary antibodies in blocking solution (5% BSA in PBS) at 4 °C overnight. The secondary antibodies were diluted in blocking solution and incubated for 1 h at room temperature and mounted with Vectashield mounting medium (Vector, Burlingame, CA). The following antibodies were used in experiments: GFP (Aves; 1:400); PSD95 (Invitrogen; 1:200); Synaptophysin (Sigma; 1:200); Homer1 (Synaptic Systems; 1:500); Synapsin (Synaptic Systems; 1:500); Neuron dendritic morphology was visualized by GFP/DsRed fluorescence in transfection experiments. Neurons were randomly chosen. Images were acquired on the ZEISS LSM880 confocal system with a Plan-Apochromat 63× NA 1.4 oil differential interference contrast objective lens. Quantification was performed with Imaris software (Bitplane Inc., Zurich, Switzerland).

For immunostaining of brain slices, mice were anesthetized and perfused with ice-cold PBS followed by 4% (wt/vol) paraformaldehyde. Dissected mouse brain samples were post-fixed in 4% (wt/vol) paraformaldehyde for 24 h, then washed by PBS, and stored in 30% (wt/vol) sucrose solution at 4 °C. About 30 µm brain slices were coronally sectioned through a cryostat. For immunofluorescence, sections were permeabilized with 0.3% Triton X-100 in PBS for 30 min at room temperature, blocked with 5% (vol/vol) BSA in PBS for 1 h at room temperature. After blocking, sections were incubated with the primary antibody in blocking buffer at 4 °C overnight, followed by incubation with secondary antibodies for 1 h at room temperature and mounted with Vectashield mounting medium (Vector, Burlingame, CA). The following antibodies were used in tissue staining: PSD95 (Invitrogen; 1:1000); Synaptophysin (Sigma, St Louis, MO, USA; 1:1000); Homer1 (Synaptic Systems, Goettingen, Germany; 1:1000); Synapsin (Synaptic Systems, 1:1000).

To quantify the number of dendritic protrusions, Z-stacked images were converted to maximal projection images and analyzed using ImageJ (NIH, USA). The length of secondary dendrite was measured by using ImageJ and the number of each type of dendritic protrusions in the given dendrite was manually counted. Two to three dendrites from each cell were analyzed. Colocalized pre- and postsynaptic puncta, as well as axonal Synaptotagmin 1 and vGlut1 puncta were analyzed and 3D reconstructed by using the “spot” function of Imaris software (Bitplane Inc., Zurich, Switzerland).

### Synaptotagmin 1 labeling in living cells

Exocytosed synaptic vesicles were labeled according to the previous report.^48^ Cortical neuronal cultures were incubated at 37 °C for 20 min with a monoclonal antibody against the luminal domain of Synaptotagmin 1 (Synaptic Systems, Goettingen, Germany; 1:200) diluted in culture medium. Neurons were washed with PBS to remove unbounded antibody, fixed, and immunostaining with an antibody against vGlut1 (Synaptic Systems; 1:500). Images were acquired on the ZEISS LSM880 confocal system. Axonal Synaptotagmin 1 and vGlut1 puncta were 3D reconstructed and colocalization were calculated by using the “spot” function of Imaris software (Bitplane Inc., Zurich, Switzerland).

### ATP/ADP ratio analysis

To quantify the ratiometric signal of PercevalHR, the fluorescent intensity of 488 and 405 nm was analyzed by using ImageJ.^30^ The pseudo-color images reflecting the F488/F405 intensity were generated by using ImageJ.

### Crude synaptosome isolation

Hippocampal and cortical tissues were homogenized in ice-cold crude synaptosome isolation buffer (1 mM MgCl_2_, 5 mM HEPES, 0.5 mM CaCl_2_, 0.32 M sucrose). Homogenized samples were centrifuged at 800×*g* for 10 min at 4 °C. Supernatants were transferred and centrifuged at 13,800×*g* for 10 min to get the pellet fraction that is a crude synaptosome.

### Immunoblotting

Immunoblotting was performed as previously described.^49^ The following antibodies were used for this study: Phospho-Drp1 (Ser616) (D9A1) (CST, Danvers, MA, USA; 1:1000); Drp1 (D6C7) (CST; 1:1000); PSD95 (CST; 1:2000); Synaptophysin (Abcam, Cambridge, UK; 1:2000); Tuj1 (Millipore, Burlington, MA, USA; 1:1000); Actin (Sigma; 1:5000); vGlut1 (Synaptic Systems; 1:500).

### Chemical LTP induction

Chemical LTP was induced by perfusion with ACSF (pH 7.4, gassed with 95% O_2_/5% CO_2_, composed of in mM: 119 NaCl, 2.5 KCl, 26.2 NaHCO_3_, 1 NaH_2_PO_4_, 5 CaCl_2_, and 11 d-glucose) containing 25 mM TEA for 15 min. For immunoblotting analysis, neurons were treated with 25 mM TEA for 2.5, 5, and 15 min. After 15 min TEA treatment, TEA was washed followed by harvesting at 15 and 45 min. For immunofluorescence analysis, neurons were treated with TEA (25 mM, TEA) or solvent (Basal) for 15 min, followed by fixation.

### Golgi staining

Mice were perfused with ice-cold PBS. Brains were dissected and immediately transferred into the Bioenno Tech superGolgi kit (Bioenno Tech, Santa Ana, CA, USA) for the Golgi-Cox method of staining in accordance with the manufacturer’s instructions. Briefly, the samples were impregnated with the potassium dichromate and mercuric chloride solution at room temperature for 10 days. After impregnation, transfer them into Post-impregnation Buffer for 2 days post-impregnation. Brains were then serially sectioned at 150 µm using a vibrating microtome and sections were collected in Collection & Mounting Buffer. Sections were mounted on adhesive microscope slides. Slices were washed in 0.01 M PBS-T for 30 min and then placed in a diluted solution C (3:5 with H_2_O) for 30 min in a closed staining jar. After that, place slides in reagent D-prepared Post-staining Buffer for 20 min in a dark area, and then wash in 0.01 M PBS-T three times, for 10 min. Air-dry the slides out of direct light. Dehydrate sections in 100% ethanol for 10 min and repeat four times. Sections were cleared in xylene three times, 10 min per time, and mounted.

### Transmission electron microscopy (TEM)

Mice were anesthetized and perfused with ice-cold PBS, followed by 2% (wt/vol) paraformaldehyde/2% (wt/vol) glutaraldehyde in 0.1 M sodium phosphate buffer. Hippocampi and cortex were removed and post-fixed in 2.5% (wt/vol) glutaraldehyde in 0.1 M sodium phosphate buffer, then immersed in osmium tetraoxide (19150, Electron Microscopy Sciences, Hatfield, USA), dehydrated in ethanol (46139, Sigma-Aldrich, St. Louis, USA), and embedded in Epon (45345, Sigma-Aldrich, St. Louis, USA). After polymerization of Epon, blocks were sectioned to generate 70 nm thick sections using a diamond knife on a microtome (Leica, Wetzlar, Germany). The sections were stained with uranyl acetate (19481, TED PELLA, Redding, USA) and lead citrate (15326, Sigma-Aldrich, St. Louis, USA). Digital images were obtained on a Tecnai G2 Spirit by FEI equipped with an Eagle 4k HS digital camera.

### Morris water maze (MWM)

The MWM was performed as previously described with minor modifications.^50^ The MWM was conducted in a circular pool (120 cm in diameter and 50 cm in height) with a featureless inner surface, filled with opaque nontoxic white dye. The water pool was placed in a dimly lit, soundproof test room surrounded by distinct extra maze cues. The pool was equally divided into four quadrants. A white platform was placed randomly in one of the quadrants and submerged 1 cm below the water surface to ensure that the platform was invisible. A pretraining day was dedicated for swim training (60 s) without the platform. This was conducted in the subsequent 5 consecutive days. During training days, 7-month-old *Drp1*^*S616A*^ KI male mice and WT control male littermates received two training sessions per day, with each session containing three trials. The drop location was changed semi-randomly between trials with the platform location fixed throughout the training process. After a mouse located the platform, it was permitted to stay on it for 10 s. A mouse was guided to the platform and placed on it for 10 s if it cannot locate the platform within 60 s. The mouse was taken to its home cage and allowed to dry up under a dim lamp after each trial. The time interval between each session was 30 min. Probe trials were conducted after the last training session (1 and 24 h after). During the probe trial, the platform was removed. Mice were allowed to swim for 60 s. All mice were placed at the same starting point, which was the farthest dropping location of the platform. During all trials, latency time, swim paths, crossing times, and percentage of time spent in each quadrant were recorded using a video camera-based Top-scan System (Clever Sys, Inc).

### Fear conditioning

On day 1, 9-month-old mice were allowed to explore the test chamber (Clever Sys, Inc) for 2 min. On day 2, mice were placed in the same test chamber for 3 min, followed by exposed to the conditioned stimulus (CS; a 2800 Hz, lasting for 30 s and flash lasting for 3 s) co-terminated with the unconditioned stimulus (1.0 mA of continuous 1 s footshock). After two cycles, mice were detained for 3 min before being returned to the home cage.

To test for contextual fear memory, mice were placed in the training chamber and monitored for 3 min at 1 or 24 h after training. To test for cued fear memory, mice were placed in a novel chamber at 1 or 24 h post-training, and exposed to CS at 3 min after they were introduced into the chamber. Freezing was defined as the complete immobility of the animal, except for respiratory movement.

### Statistics

Student’s *t*-test was used to determine the significance of the difference between the two groups. Statistical significance between each group with a control group was analyzed using one-way ANOVA followed by Dunnett’s multiple comparison test. All error bars indicate SEM. *p < 0.05; **p < 0.01; ***p < 0.001; ****p < 0.0001.

## Supplementary information

Supplementary Materials

## Data Availability

All data are available in the main text or supplementary materials. Further information and requests for resources and reagents should be directed to and will be fulfilled by the lead contact, Zhuohua Zhang (zhangzhuohua@sklmg.edu.cn).

## Materials availability

All materials are available in the main text or supplementary materials. Further information and requests for resources and reagents should be directed to and will be fulfilled by the lead contact, Zhuohua Zhang (zhangzhuohua@sklmg.edu.cn).

## References

[1] Mattson MP, Gleichmann M, Cheng A (2008). Mitochondria in neuroplasticity and neurological disorders. Neuron.

[2] Flippo KH, Strack S (2017). Mitochondrial dynamics in neuronal injury, development and plasticity. J. Cell Sci..

[3] Dorn GW (2019). Evolving concepts of mitochondrial dynamics. Annu. Rev. Physiol..

[4] Sheng ZH (2014). Mitochondrial trafficking and anchoring in neurons: new insight and implications. J. Cell Biol..

[5] Li Z, Okamoto K, Hayashi Y, Sheng M (2004). The importance of dendritic mitochondria in the morphogenesis and plasticity of spines and synapses. Cell.

[6] Divakaruni SS (2018). Long-term potentiation requires a rapid burst of dendritic mitochondrial fission during induction. Neuron.

[7] Fu ZX (2017). Dendritic mitoflash as a putative signal for stabilizing long-term synaptic plasticity. Nat. Commun..

[8] Ishihara N (2009). Mitochondrial fission factor Drp1 is essential for embryonic development and synapse formation in mice. Nat. Cell Biol..

[9] Shields LY (2015). Dynamin-related protein 1 is required for normal mitochondrial bioenergetic and synaptic function in CA1 hippocampal neurons. Cell Death Dis..

[10] Chandra R (2017). Drp1 mitochondrial fission in D1 neurons mediates behavioral and cellular plasticity during early cocaine abstinence. Neuron.

[11] Westermann B (2010). Mitochondrial fusion and fission in cell life and death. Nat. Rev. Mol. Cell Biol..

[12] Taguchi N, Ishihara N, Jofuku A, Oka T, Mihara K (2007). Mitotic phosphorylation of dynamin-related GTPase Drp1 participates in mitochondrial fission. J. Biol. Chem..

[13] Cribbs JT, Strack S (2007). Reversible phosphorylation of Drp1 by cyclic AMP-dependent protein kinase and calcineurin regulates mitochondrial fission and cell death. EMBO Rep..

[14] Dickey AS, Strack S (2011). PKA/AKAP1 and PP2A/Bbeta2 regulate neuronal morphogenesis via Drp1 phosphorylation and mitochondrial bioenergetics. J. Neurosci..

[15] Godoy JA (2014). Wnt-5a ligand modulates mitochondrial fission-fusion in rat hippocampal neurons. J. Biol. Chem..

[16] Scarffe LA, Stevens DA, Dawson VL, Dawson TM (2014). Parkin and PINK1: much more than mitophagy. Trends Neurosci..

[17] Poole AC (2008). The PINK1/Parkin pathway regulates mitochondrial morphology. Proc. Natl Acad. Sci. USA.

[18] Deng H, Dodson MW, Huang H, Guo M (2008). The Parkinson’s disease genes pink1 and parkin promote mitochondrial fission and/or inhibit fusion in Drosophila. Proc. Natl Acad. Sci. USA.

[19] Liu W (2011). Pink1 regulates the oxidative phosphorylation machinery via mitochondrial fission. Proc. Natl Acad. Sci. USA.

[20] Yang Y (2008). Pink1 regulates mitochondrial dynamics through interaction with the fission/fusion machinery. Proc. Natl Acad. Sci. USA.

[21] Dagda RK (2009). Loss of PINK1 function promotes mitophagy through effects on oxidative stress and mitochondrial fission. J. Biol. Chem..

[22] Yu W, Sun Y, Guo S, Lu B (2011). The PINK1/Parkin pathway regulates mitochondrial dynamics and function in mammalian hippocampal and dopaminergic neurons. Hum. Mol. Genet..

[23] Han H (2020). PINK1 phosphorylates Drp1(S616) to regulate mitophagy-independent mitochondrial dynamics. EMBO Rep..

[24] Peters A, Kaiserman-Abramof IR (1970). The small pyramidal neuron of the rat cerebral cortex. The perikaryon, dendrites and spines. Am. J. Anat..

[25] Yuste R, Bonhoeffer T (2004). Genesis of dendritic spines: insights from ultrastructural and imaging studies. Nat. Rev. Neurosci..

[26] Bourne JN, Harris KM (2008). Balancing structure and function at hippocampal dendritic spines. Annu. Rev. Neurosci..

[27] Gu QH (2015). miR-26a and miR-384-5p are required for LTP maintenance and spine enlargement. Nat. Commun..

[28] Huang YY, Malenka RC (1993). Examination of TEA-induced synaptic enhancement in area CA1 of the hippocampus: the role of voltage-dependent Ca2+ channels in the induction of LTP. J. Neurosci..

[29] Huber KM, Mauk MD, Kelly PT (1995). Distinct LTP induction mechanisms: contribution of NMDA receptors and voltage-dependent calcium channels. J. Neurophysiol..

[30] Tantama M, Martinez-Francois JR, Mongeon R, Yellen G (2013). Imaging energy status in live cells with a fluorescent biosensor of the intracellular ATP-to-ADP ratio. Nat. Commun..

[31] Keil U (2006). Piracetam improves mitochondrial dysfunction following oxidative stress. Br. J. Pharm..

[32] Rangaraju V, Lauterbach M, Schuman EM (2019). Spatially stable mitochondrial compartments fuel local translation during plasticity. Cell.

[33] Ma H, Cai Q, Lu W, Sheng ZH, Mochida S (2009). KIF5B motor adaptor syntabulin maintains synaptic transmission in sympathetic neurons. J. Neurosci..

[34] Lewis TL, Kwon SK, Lee A, Shaw R, Polleux F (2018). MFF-dependent mitochondrial fission regulates presynaptic release and axon branching by limiting axonal mitochondria size. Nat. Commun..

[35] Youle RJ, Narendra DP (2011). Mechanisms of mitophagy. Nat. Rev. Mol. Cell Biol..

[36] Harris JJ, Jolivet R, Attwell D (2012). Synaptic energy use and supply. Neuron.

[37] Hernandez CJ, Baez-Becerra C, Contreras-Zarate MJ, Arboleda H, Arboleda G (2019). PINK1 silencing modifies dendritic spine dynamics of mouse hippocampal neurons. J. Mol. Neurosci..

[38] Lutz AK (2009). Loss of parkin or PINK1 function increases Drp1-dependent mitochondrial fragmentation. J. Biol. Chem..

[39] Cho B (2014). CDK5-dependent inhibitory phosphorylation of Drp1 during neuronal maturation. Exp. Mol. Med..

[40] Valente EM (2004). Hereditary early-onset Parkinson’s disease caused by mutations in PINK1. Science.

[41] Xiong H (2009). Parkin, PINK1, and DJ-1 form a ubiquitin E3 ligase complex promoting unfolded protein degradation. J. Clin. Invest.

[42] Yuan CL, Hu YC (2017). A transgenic core facility’s experience in genome editing revolution. Adv. Exp. Med. Biol..

[43] Scott MA, Hu YC (2019). Generation of CRISPR-edited rodents using a piezo-driven zygote injection technique. Methods Mol. Biol..

[44] Viesselmann, C., Ballweg, J., Lumbard, D. & Dent, E. W. Nucleofection and primary culture of embryonic mouse hippocampal and cortical neurons. *J. Vis. Exp.* **47**, 2373 (2011). 10.3791/2373 PMC3182630 21304471

[45] Yang X (2018). A novel mechanism of memory loss in Alzheimer’s disease mice via the degeneration of entorhinal-CA1 synapses. Mol. Psychiatry.

[46] Deng S (2020). Regulation of recurrent inhibition by asynchronous glutamate release in neocortex. Neuron.

[47] Li X (2019). Presynaptic endosomal cathepsin D regulates the biogenesis of GABAergic synaptic vesicles. Cell Rep..

[48] Kraszewski K (1995). Synaptic vesicle dynamics in living cultured hippocampal neurons visualized with CY3-conjugated antibodies directed against the lumenal domain of synaptotagmin. J. Neurosci..

[49] Zhang T (2016). BNIP3 protein suppresses PINK1 kinase proteolytic cleavage to promote mitophagy. J. Biol. Chem..

[50] Chiu SL (2017). GRASP1 regulates synaptic plasticity and learning through endosomal recycling of AMPA receptors. Neuron.

