# Supplementary material for: PINK1-mediated Drp1S616 phosphorylation modulates synaptic development and plasticity via promoting mitochondrial fission
Source: Signal Transduct Target Ther. 2022 Apr 15;7:103. doi: 10.1038/s41392-022-00933-z (PMC9010405; doi:10.1038/s41392-022-00933-z)
Supplement: Supplementary file 1 — Supplementary Materials [file 41392_2022_933_MOESM1_ESM.docx]

**Supplementary Materials for**

**PINK1-mediated Drp1^S616^ phosphorylation modulates synaptic development and plasticity via promoting mitochondrial fission**

Qingtao Gao^1, 9^, Runyi Tian^1, 9^, Hailong Han^1^, Jesse Slone^2^, Caifang Wang^1^, Xiao Ke^3, 4^, Tongmei Zhang^3, 4^, Xiangyu Li^8^, Yuhong He^1^, Panlin Liao^1^, Fang Wang^5^, Ye Chen^1^, Shiqing Fu^5^, Kexuan Zhang^1^, Fangfang Zeng^1^, Yingxuan Yang^1^, Zhuo Li^8^, Jieqiong Tan^1^, Jiada Li^8^, Youming Lu^3, 4^, Taosheng Huang^2^, Zhonghua Hu^1, 6, 7, 8^*, Zhuohua Zhang^1, 5, 6, 8^*

Correspondence to: [zhangzhuohua@sklmg.edu.cn](mailto:zhangzhuohua@sklmg.edu.cn)；[huzhonghua@csu.edu.cn](mailto:huzhonghua@csu.edu.cn).

**This PDF file includes:** Supplementary Figures S1 to S6

Supplementary Figure S1.


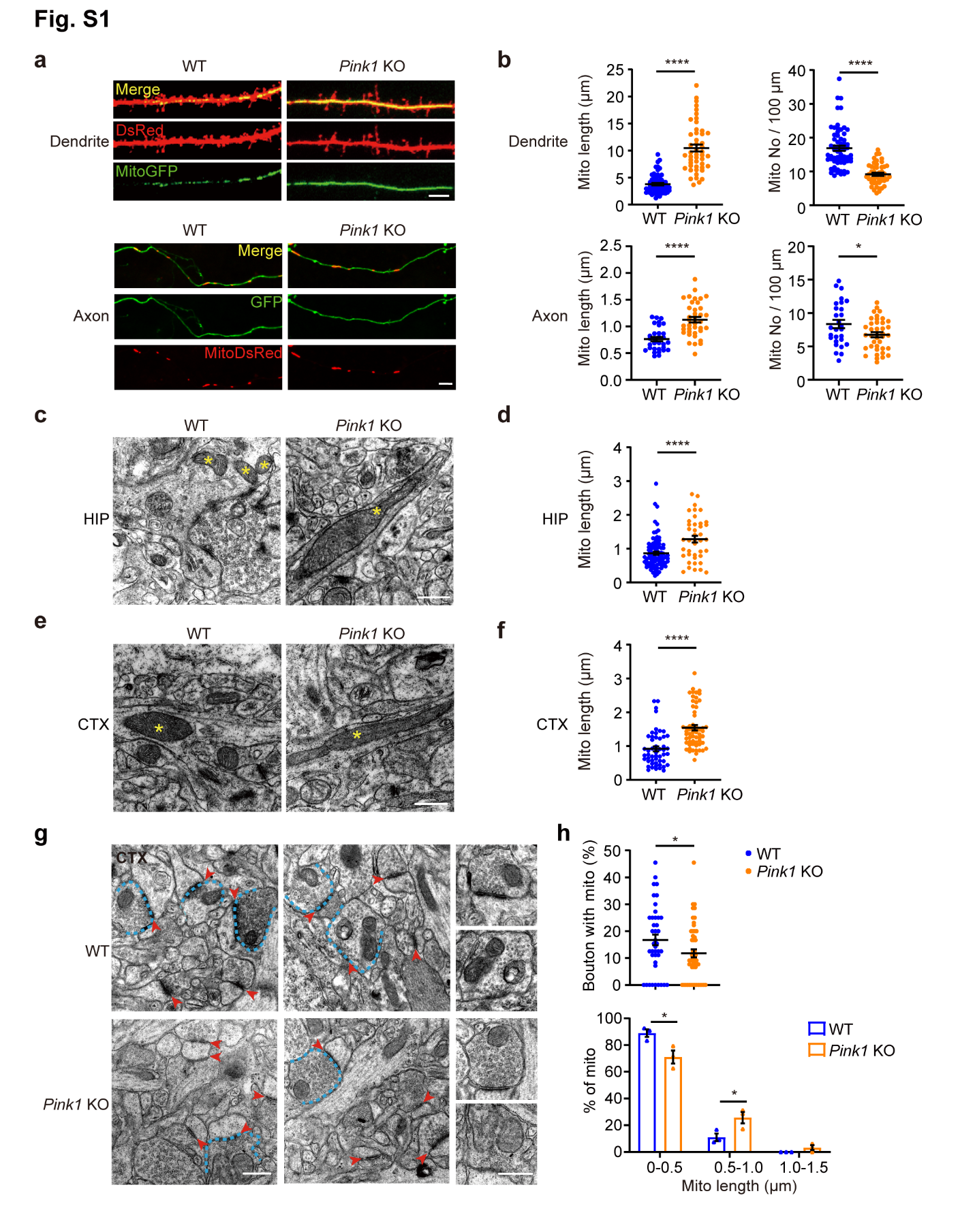
 **Supplementary Fig. S1. PINK1 promotes mitochondrial fission in neurons.**

**a**. Hippocampal neurons derived from WT or *Pink1* KO mice were transfected with plasmids encoding MitoGFP and DsRed. Representative images of dendrites and dendritic mitochondria (green) are shown in upper panel. Cortical neurons were cultured and transfected with plasmids encoding MitoDsRed and EGFP followed by fixation at DIV18. Representative images of axonal mitochondria (red) were shown in lower panel. Bar=5 μm.

**b**. Quantitative analysis of mitochondrial number (Mito No /100 μm) and mitochondrial length (Mito length) in dendrites and axons from experiments shown in **a**. n ≥ 48 dendrites and 30 axons of WT and *Pink1* KO from 3 independent experiments, respectively.

**c**, **e**. Representative TEM images of dendritic mitochondria (yellow asterisk) in WT and *Pink1* KO hippocampal neurons (c, HIP) or cortical neurons (e, CTX) are shown. Bar=0.5 μm.

**d**, **f**. Quantitative analysis of dendritic mitochondrial length from experiments shown in **c** or **e**. For **d**, n = 87 and 41 sections for WT and *Pink1* KO, respectively. For **f**, n = 52 and 60 sections for WT and *Pink1* KO, respectively.

**g**. Representative TEM images of synapses (red arrows) and presynaptic boutons with mitochondria (blue outlines) in somatosensory cortex from 8-week-old WT and *Pink1* KO mice. Images with high magnification are shown (right panels). Bar=0.5 μm.

**h**. Quantitation analysis of presynaptic bouton with mitochondria (upper panel, Bouton with mito) and mitochondrial length (lower panel). Presynaptic bouton with mitochondria: n= 41 and 50 sections from 3 WT and 3 *Pink1* KO mice, respectively. Mitochondrial length analysis: n= 3 WT mice and 3 *Pink1* KO mice with 66 mitochondria for each genotype.

^*^p < 0.05, ^****^p < 0.0001. Student’s t-test.

Supplementary Figure S2.


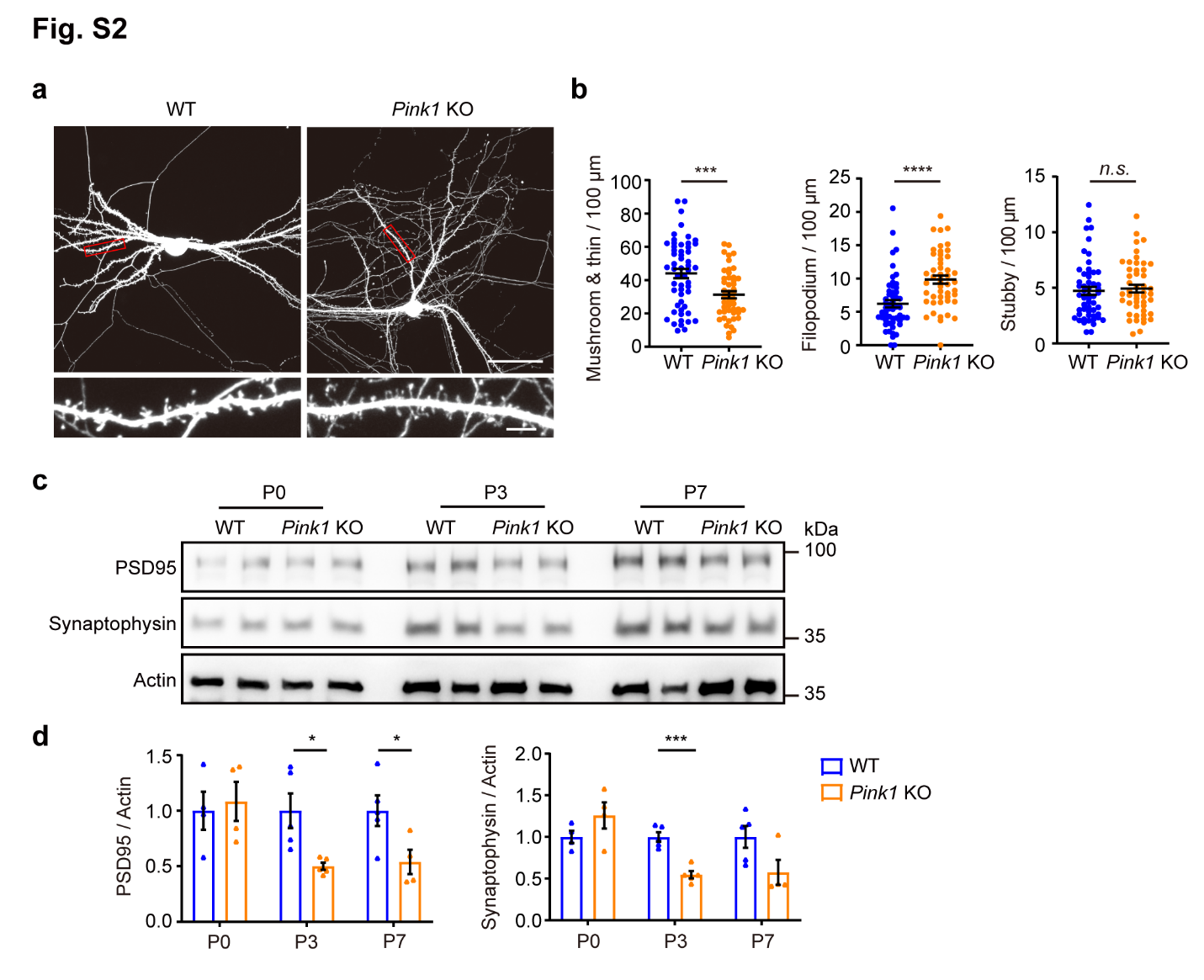


**Supplementary Fig. S2. PINK1 regulates excitatory synapse maturation**.

**a**. Hippocampal neurons (DIV 6-8) derived from WT or *Pink1* KO mice were transfected with plasmid encoding DsRed and imaged at DIV 18. Representative images of transfected neurons (upper panels, Bar=50 μm) and dendrites at a higher magnification (lower panels, Bar=5 μm).

**b**. Quantification analysis of different types of dendritic spines for experiments shown in **a**. n = 54 and 48 dendrites for WT and *Pink1* KO cultures from 3 independent experiments, respectively.

**c**. Immunoblot detection of PSD95, synaptophysin, and actin in crude synaptosome fraction extracted from cortical tissues of WT and *Pink1* KO mice at the age of P0, P3, and P7.

**d**. Quantitative analysis of PSD95 (PSD95 / Actin) and synaptophysin (Synaptophysin / Actin) for experiments shown in **c**. n= 4-5 mice/genotype.

^*^p < 0.05, ^***^p < 0.001, ^****^p < 0.0001, *n.s.* not significant. Student’s t-test.

Supplementary Figure S3.


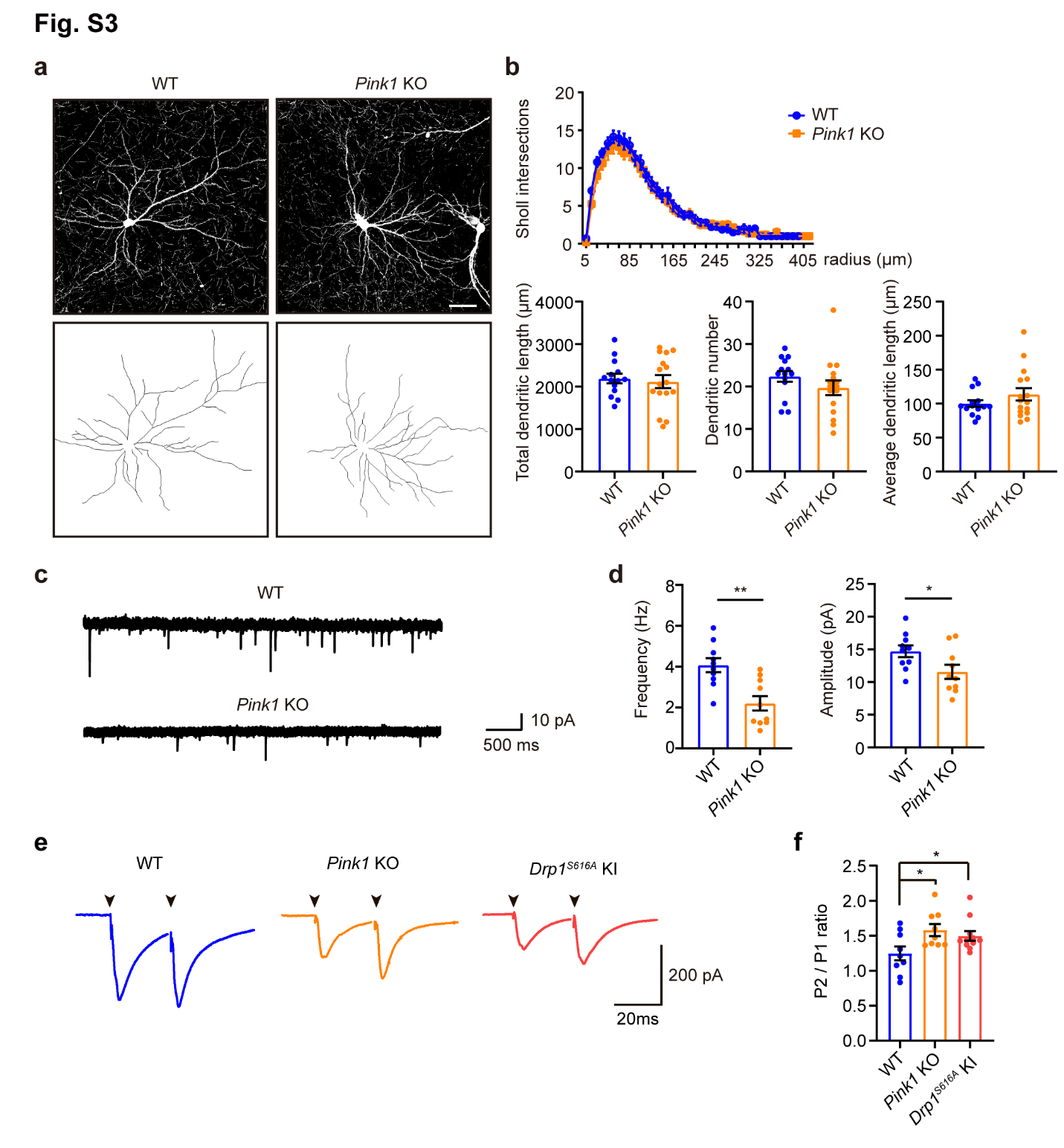


**Supplementary Fig. S3. PINK1 does not affect development of early dendrite but synaptic transmission**

**a**. Cortical neurons were transfected with EGFP construct at DIV 6-8 and imaged at DIV 18. Representative images of transfected neurons (upper panels) and reconstructed traces of WT and *Pink1* KO pyramidal neurons by Image J (lower panels) are shown. Bar=50 μm.

**b**. Quantitative analysis of intersections in **a**. Sholl-analysis indicates no differences (p > 0.05 in Student’s t-test) in dendritic length, dendric number and intersections between neurons from WT and *Pink1* KO mice. n = 14-16 neurons for each group.

**c**. Representative traces of mEPSCs recorded from hippocampal CA1 neurons of 12-week-old WT and *Pink1* KO mice.

**d**. Quantitation of mEPSCs frequencies and amplitudes for experiments shown in **c**. n = 10 cells from 3 WT and 3 Pink1 KO mice.

**e**. Sample traces showing paired-pulse facilitation increased in *Pink1* KO and *Drp1^S616A^* KI somatosensory cortex Layer 2/3 neurons. Paired-pulse facilitation was measured in whole-cell patch-clamped neurons at 20 msec intervals. The stimulation artifacts were removed.

**f**. Quantification of the ratio of second EPSC (P2) to the first EPSC (P1) in S3e. The peak amplitude of the response to the second pulse was averaged over 10 trials and divided by the averaged peak amplitude of the response to the first pulse to give a paired-pulse ratio (P2 / P1). n = 9-11 cells from 3-4 mice per genotype.

^*^p < 0.05, ^**^p < 0.01. Student’s t-test.

Supplementary Figure S4.


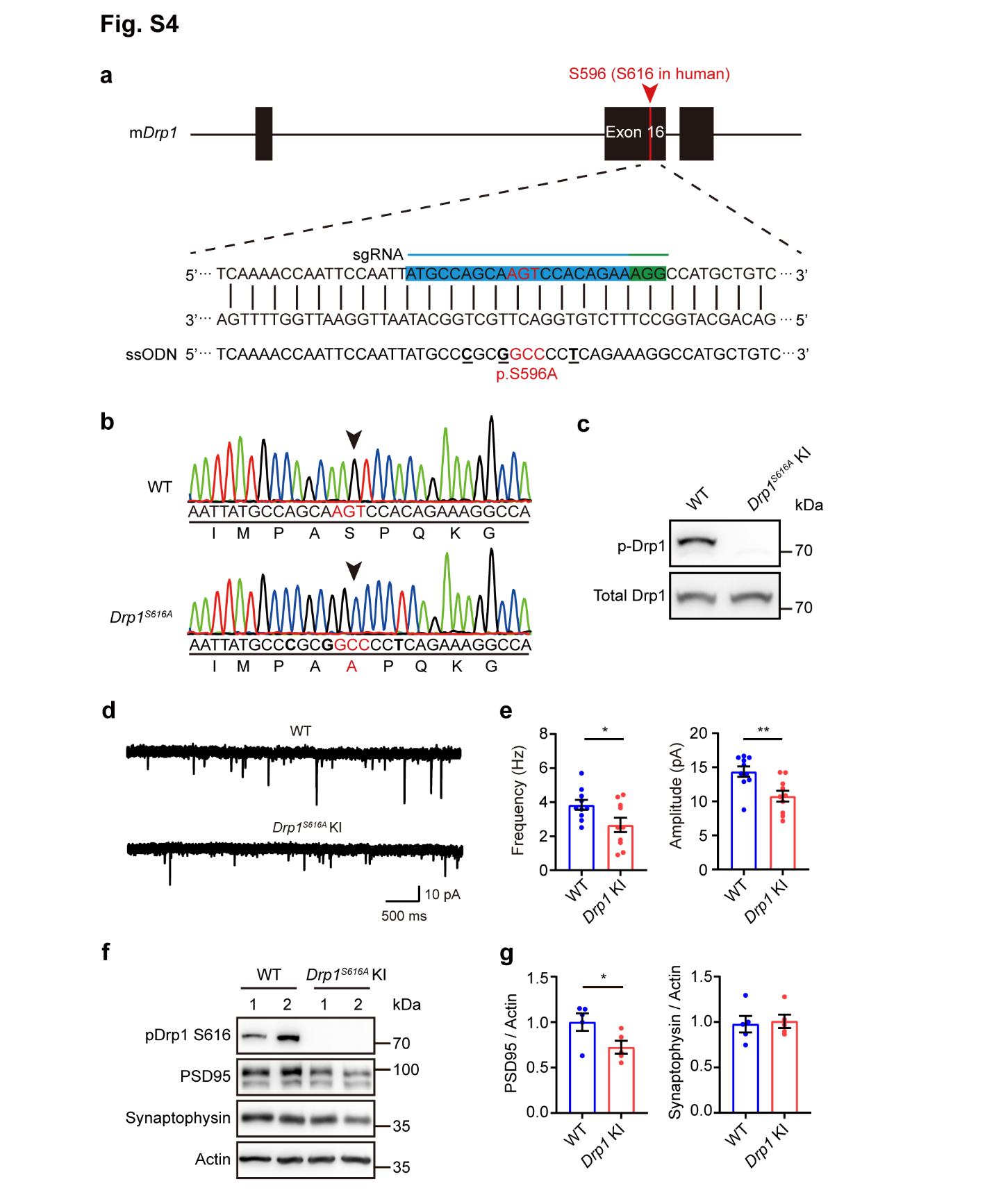


**Supplementary Fig. S4. Generation of *Drp1^S616A^* KI mice and evaluation of synaptic strength**

**a**. A schematic representation of *Drp1^S616A^* KI strategy. S596 of mouse Drp1 variant-3 (NM_001276340.1) corresponds to human Drp1 residue S616 (NM_012062.5), hereafter mouse Drp1 S596A mutation is referred as *Drp1^S616A^*. Mouse S596 is located in exon 16 of Drp1 gene (upper panel). Relative position in genomic sequence and sequences of sgRNA target (Blue), PAM sequence (green), S596 site (red) are shown (middle panel). The 125-bp ssODN donor template with a S596A mutant sequence (red) is illustrated (lower panel). Three nucleotides modified to prevent Cas9 recut are bolded and underlined.

**b**. Chromatograms of genomic DNA sequencing of wildtype (upper panel) and *Drp1^S616A^* KI mice (lower panel) are shown. Position to introduce *Drp1^S616A^* mutation are highlighted (red). The coding amino acids are shown underneath nucleic acid sequences.

**c**. Immunodetection of phosphor-Drp1^S616^ (pDrp1^S616^) and total Drp1 in liver lysates of WT and *Drp1^S616A^* KI mice. Note that *Drp1^S616A^* KI does not change the total Drp1 while it abolishes Drp1^S616^ phosphorylation.

**d**. Representative traces of mEPSCs recorded from hippocampal CA1 neurons of 12- week-old WT and *Drp1^S616A^* KI mice.

**e**. Quantitation of mEPSCs frequencies and amplitudes for experiments shown in **d**. n = 10 cells from 3 WT and 3 *Drp1^S616A^* KI mice.

**f**. Immunoblots of PSD95 and synaptophysin of crude synaptosome fraction extracted from WT and *Drp1^S616A^* KI cortical tissues. Actin is detected as loading control.

**g**. Quantitative analysis of synaptic proteins for experiments shown in **f**. n = 5 mice per genotype. ^*^p < 0.05, ^**^p < 0.01, Student’s t-test.

**Supplementary Figure S5.**


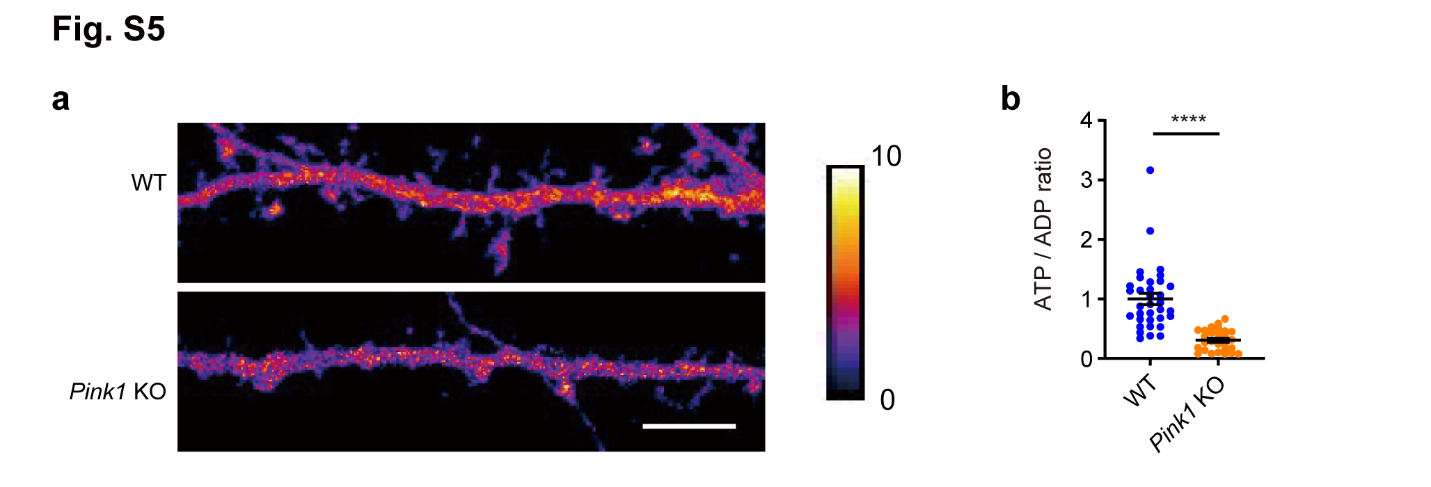


**Supplementary Fig. S5. Reduced ATP / ADP ratio at dendrites of *Pink1* KO neurons**

Cortical neuronal cultures (DIV 14) derived from WT or *Pink1* KO mice were transfected with a PercevalHR construct using Lipofectamine 2000. Neurons were imaged at DIV 18. Representative pseudo-color images of transfected neurons are shown (**a**). Bar=5 μm. Quantitation of ratiometric mean intensity of F488nm / F405nm (ATP / ADP ratio) in dendrites is presented (**b**). n = 34 and 28 dendrites for WT and *Pink1* KO cultures from 2 independent experiments, respectively. ^****^p < 0.0001, Student’s t-test.

**Supplementary Figure S6.**


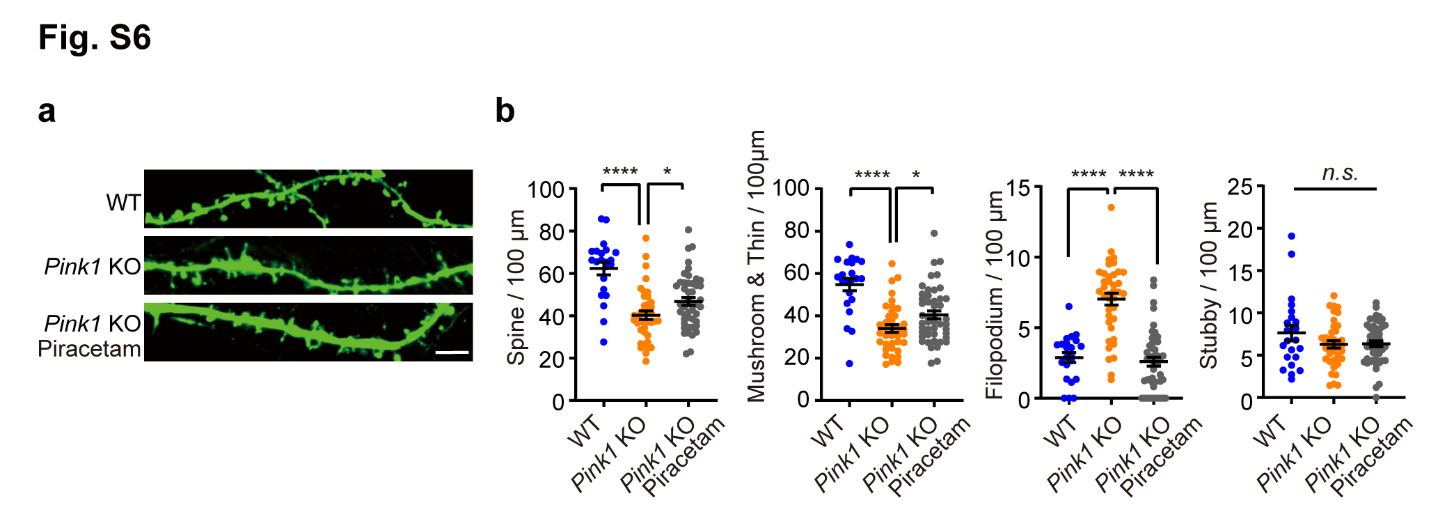


**Supplementary Fig. S6. Piracetam treatment rescues spine defects in *Pink1* KO neurons**

Cortical neuronal cultures (DIV 14) derived from WT or *Pink1* KO mice were transfected with a plasmid encoding EGFP followed by a treatment with either piracetam (1mM) or vehicle at the same day. Cells were fixed and analyzed 4 days later. Representative images of dendritic spines of transfected neurons are shown (**a**). WT: neurons derived from WT mice. *Pink1* KO: neurons derived from *Pink1* KO mice. *Pink1* KO Piracetam: neurons derived from *Pink1* KO mice that are treated with piracetam. Bar=5 μm. Quantitative analysis of different types of dendritic spines for the experiments shown in **a** (**b**). n= 22-47 dendrites / condition from 2 independent experiments, respectively. ^*^p < 0.05, ^****^p < 0.0001, *n.s.* not significant. One-way ANOVA followed by Dunnett’s multiple comparison test.
